# *Alternaria alternata* Mycotoxins Activate the Aryl Hydrocarbon Receptor and Nrf2-ARE Pathway to Alter the Structure and Immune Response of Colon Epithelial Cells

**DOI:** 10.1021/acs.chemrestox.1c00364

**Published:** 2022-04-11

**Authors:** Julia Groestlinger, Veronika Spindler, Gudrun Pahlke, Michael Rychlik, Giorgia Del Favero, Doris Marko

**Affiliations:** † Department of Food Chemistry and Toxicology, Faculty of Chemistry, University of Vienna, Währinger Straße 38, 1090 Vienna, Austria; ‡ Chair of Food Analytical Chemistry, Technical University of Munich, Maximus-von-Imhof-Forum 2, 85354 Freising, Germany; § Core Facility Multimodal Imaging, Faculty of Chemistry, University of Vienna, Währinger Straße 38, 1090 Vienna, Austria

## Abstract

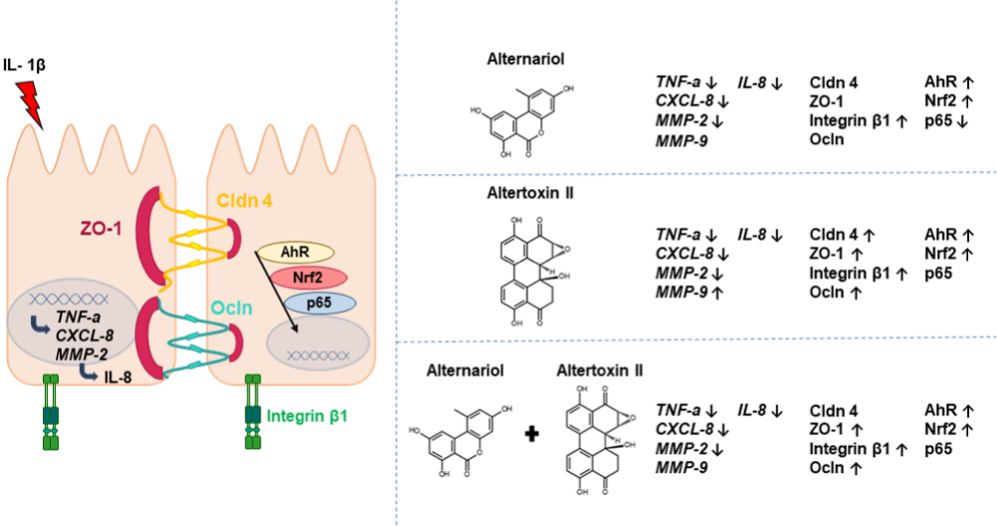

After ingestion of food commodities, the gastrointestinal tract (GIT) poses the first barrier against xenobiotics and pathogens. Therefore, it is regularly confronted with external stressors potentially affecting the inflammatory response and the epithelial barrier. *Alternaria* mycotoxins such as alternariol (AOH) and altertoxin II (ATX-II) are frequently occurring food and feed contaminants that are described for their immunomodulatory capacities. Hence, this study aimed at exploring the effect of AOH and ATX-II as single compounds or binary mixtures on the immune response and epithelial homeostasis in noncancerous colon epithelial cells HCEC-1CT. Both toxins suppressed mRNA levels of proinflammatory mediators interleukin-8 (IL-8), tumor necrosis factor α (TNF-α), and secretion of IL-8, as well as mRNA levels of the matrix metallopeptidase 2 (MMP-2). Binary combinations of AOH and ATX-II reduced the response of the single toxins. Additionally, AOH and ATX-II modified immunolocalization of transmembrane proteins such as integrin β1, zona occludens 1 (ZO-1), claudin 4 (Cldn 4), and occludin (Ocln), which support colonic tissue homeostasis and intestinal barrier function. Moreover, the cellular distribution of ZO-1 was affected by ATX-II. Mechanistically, these effects could be traced back to the involvement of several transcription factors. AOH activated the nuclear translocation of the aryl hydrocarbon receptor (AhR) and the nuclear factor erythroid 2-related factor 2 (Nrf2), governing cell metabolic competence and structural integrity. This was accompanied by altered distribution of the NF-κB p65 protein, an important regulator of inflammatory response. ATX-II also induced AhR and Nrf2 translocation, albeit failing to substantiate the effect of AOH on the colonic epithelium. Hence, both toxins coherently repress the intestinal immune response on the cytokine transcriptional and protein levels. Furthermore, both mycotoxins affected the colonic epithelial integrity by altering the cell architecture.

## Introduction

The gastrointestinal tract (GIT) serves as the prime barrier against environmental and foodborne stressors. To serve this purpose, structure and metabolic competence are necessary and are obtained thanks to the intestinal mucosal barrier composed of a mucus layer and the intestinal epithelium. Upon disturbance of physiological homeostasis, inflammatory cascades contribute to restoring the epithelial barrier function. Thus, intestinal epithelial cells (IECs) communicate with immune cells and participate in the local immune response.^1,2^ With the onset of inflammatory response, pro- and anti-inflammatory cytokines are strictly orchestrated. Subsequently, tightly regulated signaling between epithelial and immune cells within the intestinal tissue aims to resolve the inflammation, restore tissue homeostasis, and support epithelial restitution. Any disruption of these complex and manifold interactions may result in chronic inflammation or continuously damaged tissue.^3,4^

In this respect, the proinflammatory cytokine interleukin-1β (IL-1β) is known to participate in inflammatory response mechanisms involved in acute and chronic inflammatory diseases. It was suggested to possibly activate both the transcription factor nuclear factor “kappa-light-chain-enhancer” of activated B-cells (NF-κB) and the mitogen-activated protein kinases (MAPKs).^5^ Initially, activation of NF-κB signaling is part of the successful elimination of external stressors via proinflammatory cytokines such as interleukin-8 (IL-8) or tumor necrosis factor α (TNF-α). Furthermore, the NF-κB pathway was previously implied to contribute to tissue remodeling via upregulation of matrix metallopeptidases (MMPs) and intestinal immune homeostasis.^3^ Nonetheless, if dysregulated or constitutively activated, it contributes to the pathogenesis of chronic inflammatory diseases affecting the gastrointestinal tract.^6^

Obviously, inflammation is regulated at multiple levels and via intersecting pathways: for example, activated aryl hydrocarbon receptor (AhR) was previously reported to interact with players of immune response cascades,^7^ including transcription factors of the canonical and noncanonical NF-κB signaling.^8^ In addition, the binding of endogenous and exogenous ligands to the AhR enhances the transcription and expression of phase-I-metabolism enzymes, such as cytochromes of the CYP 1 family, as well as phase-II-metabolism enzymes.^9^ Importantly, for the response to external stressors, AhR activation was reported to exert also antioxidative potential by inducing the Nrf2-ARE pathway.^8^ Similar to AhR, Nrf2 also constitutes an important defense mechanism for intestinal cells: oxidative stress or electrophiles promote the release of Nrf2 from its binding partner KELCH-like ECH-associated protein-1 (Keap 1) protein and activate the transcription factor. Upon nuclear translocation, Nrf2 dictates the transcription of genes encoding for phase-I and phase-II-metabolizing enzymes.^10^ In addition, oxidative stress is known to also induce proinflammatory signaling pathways including NF-κB; however, activation of the Nrf2-ARE pathway, in turn, was reported to suppress NF-κB signaling by various means.^11,12^ Among others, the molecular events downstream of these two transcription factors were reported to be interconnected, particularly at the activation of the NF-κB protein p65 (RelA).^11,13^

During inflammation, the epithelial barrier integrity is prone to disruption; therefore, epithelia pose highly dynamic structures to allow for subsequent reconstitution as part of inflammation resolution.^3^ These processes involve well-balanced membrane proteins ensuring proper cell polarity to maintain the epithelial structure, such as integrins, and tight junction proteins, such as claudins (Cldn).^14^ Nrf2 activation was recently discussed to reinstate intestinal barrier integrity via tight junction proteins such as occludin (Ocln), zona occludens (ZO) proteins, and claudins,^15^ while AhR activation was shown to alter their localization upon TNF-α/interferon γ (IFN-γ)-induced barrier disruption.^16^ Besides, the Nrf2-ARE and NF-κB pathways are known to be interconnected in wound-healing processes after acute inflammation via regulation of various MMPs involved in tissue remodeling.^17^

**Figure 1:**
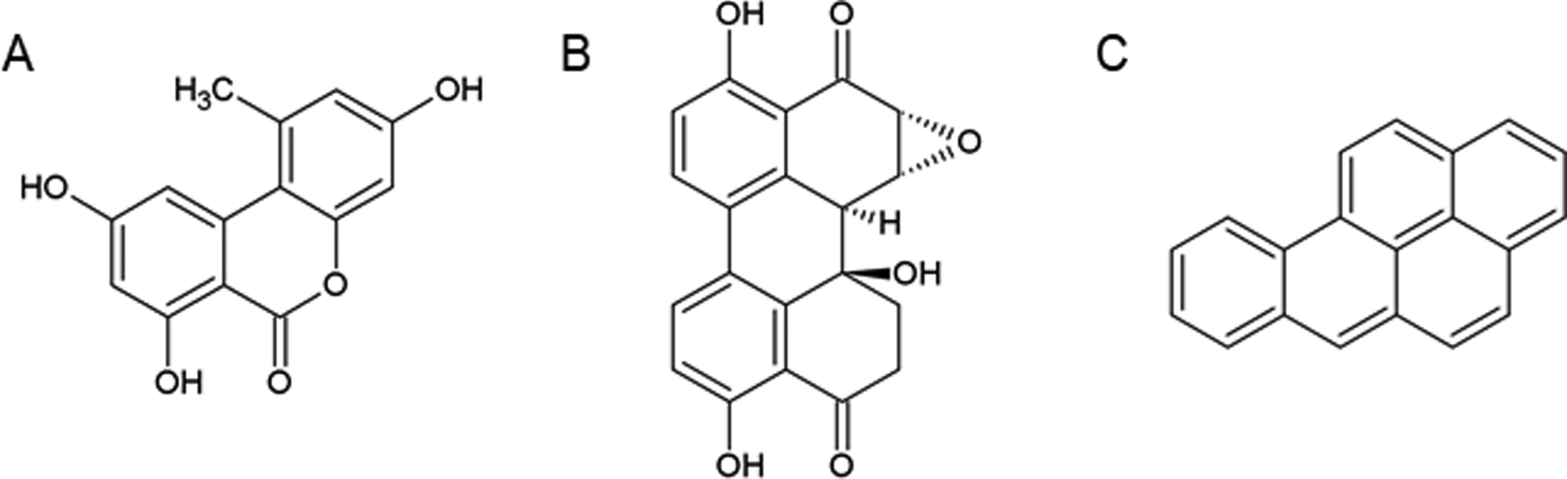
Chemical structures of the two mycotoxins alternariol (AOH) (A) and altertoxin II (ATX-II) (B) and the AhR agonist benzo[*a*]pyrene (B[*a*]P) (C).

Dietary intake of xenobiotics is known to impact the intestinal immune response and epithelial barrier function.^1,18^ Among the food contaminants potentially occurring in the diet, toxic fungal secondary metabolites play an emerging role. Amidst these, chemically diverse toxins produced by *Alternaria alternata* fungi are regularly reported to occur in food and feed; however, they are scarcely regulated in food commodities.^19^*Alternaria spp*. are known to adapt to exogenous parameters with respect to growth, germination, and production of mycotoxins.^20^ Numerous *Alternaria* toxins have recently gained attention due to their occurrence in a great variety of foodstuff, which is accompanied by bioavailability and recurrence in human body fluids reported in biomonitoring studies.^21,22^ Hence, the human GIT is prone to be exposed to varying compositions and concentrations of mixtures of *Alternaria* secondary metabolites. Among the secondary metabolites of *Alternaria spp*., the dibenzo-α-pyrone alternariol (AOH, Figure 1A) was previously described to suppress LPS-induced TNF-α secretion and gene expression in differentiated THP-1-derived macrophages.^23^ Moreover, AOH could inhibit cell proliferation via cell cycle arrest in the G2/M phase *in vitro*,^24^ induce ROS production in murine macrophage cells RAW 264.7,^25^ and exhibit estrogenic potential toward endometrial adenocarcinoma Ishikawa cells.^26^ Another secondary metabolite produced by *Alternaria* species, the perylene quinone derivative altertoxin II (ATX-II, Figure 1B), was recently shown to exhibit immunomodulatory potential toward THP-1-derived macrophages targeting the NF-κB pathway, while concurrently inducing mitochondrial superoxide generation.^27^ Besides, it has been demonstrated to exert cytotoxic and genotoxic capacities,^28^ impact cell membrane properties,^29^ and activate the Nrf2-ARE pathway.^30^ Furthermore, continuous investigations on mycotoxin co-occurrence in food and feed urge the need to pursue combinatory studies on relevant physiological and pathophysiological endpoints.^19^ A recent study revealed combined *Alternaria* toxins to interact toward the activation of the AhR signaling pathway in human breast cancer cells MCF-7. The binary mixture of ATX-II and AOH showed antagonistic interactive potential at low concentrations that turned into a synergistic interaction at higher, yet noncytotoxic, concentrations.^31^

Considering these interactions, this study investigated the potential of two *Alternaria* toxins AOH and ATX-II to exert immunomodulatory effects on noncancerous colonic epithelial cells HCEC-1CT. For this purpose, the colon cells were exposed to AOH and ATX-II in various concentrations, as well as a binary mixture in a 10:1 ratio with subsequent proinflammatory stimulation, applying IL-1β. The toxins’ effects were explored by conducting a quantitative real-time polymerase chain reaction (qRT-PCR) and Bio-Plex multiplex immunoassay measurements investigating cytokines’ mRNA and protein secretion levels. Furthermore, the underlying signaling pathways, as well as implications on epithelial barrier function, were examined via immunofluorescence staining experiments. Thus, the present work was designed to investigate the potential of *Alternaria* toxins to affect key effectors of the intestinal barrier maintenance from transcription factors to proteins necessary for structural integrity.

## Materials and Methods

### Chemicals and Reagents for Experiments

Cell culture media and respective supplements were obtained from Gibco Thermo Fisher Scientific (Waltham, MA) and Szabo Scandic (Vienna, Austria). CellTiter-Blue (CTB) 10× concentrate was purchased from Promega (Waldorf, Germany), sulforhodamine B (SRB), AOH (96%), dexamethasone (Dex), B[*a*]P, and CH223191 (CH-22) were obtained from Sigma Aldrich Chemie GmbH & Co (Steinheim, Germany). Invitrogen CyQuant LDH Cytotoxicity Assay Kit was bought from Thermo Fisher Scientific (Waltham, MA). ATX-II was acquired from rice infected with *A. alternata* spores in-house as previously published.^28^ Triton X, dimethylsulfoxide (DMSO), formaldehyde solution (FA), trichloroacetic acid (TCA), and ROTI Mount FluorCare DAPI solution were bought from Carl Roth (Karlsruhe, Germany). Maxwell 16 LEV simplyRNA Purification Kits were bought from Promega (Walldorf, Germany), QuantiTect Primer Assays, QuantiTect Reverse Transcription Kit, and QuantiTect SYBR Green Master Mix were purchased from Qiagen (Hilden, Germany). Bio-Plex Multiplex Immunoassay reagents were purchased at Bio-Rad (Vienna, Austria) (Cat. nr.: 171B5008M, 171B5026M, 171304090M, 12007919). Recombinant human IL-1β was obtained from InvivoGen (San Diego, CA). Antibodies for immunofluorescence experiments were obtained from Abcam (Cambridge, U.K.): anti-AhR (ab84833), anti-NF-κB p65 (ab32536), anti-Nrf2 (ab89443) anti-ZO-1 (ab190085), anticlaudin 4 (Cldn 4) (ab53156), and antioccludin (ab242202). Plastic labware was bought at Sarstedt AG & Co (Nuembrecht, Germany), and microscopy slides at ibidi (Graefeling, Germany).

### Cell Culture and Experimental Layout

The epithelial nontumorigenic immortalized cell line HCEC-1CT^32^ was kindly provided by Professor Jerry W. Shay (Ut Southwestern Medical Center, Dallas, TX) and cultured as previously described.^29^ Cells were maintained in a humidified incubator (95% humidity) at 37 °C and 5% CO_2_ and regularly tested for mycoplasm. Subconfluent cells were passaged every 3–4 days. HCEC-1CT cells were seeded at a density of 14 000 cells/cm^2^ for cell viability testing and PCR experiments and at a density of 10 000 cells/cm^2^ for immunofluorescence staining experiments and grown for 48 h prior to incubations. Different incubation workflows for all experiments are depicted in Supporting Information Figure S2. Accounting for all experiments, however, is the following scheme: toxins (or control conditions, excluding IL-1β control) were incubated for 2 h prior to inflammatory stimulation (IL-1β, if applicable). For experimental setups including the AhR antagonist CH-22,^33^ it was preincubated prior to toxins for 1 h. Toxin concentration ranges applied in this study were based on prior data on sensitivity in colonic cell lines yet ensuring rather subcytotoxic conditions to dissect the toxins’ effects from cytotoxicity.^30,34^ Reported contamination ranges for AOH lie between 2.5 μg/kg (for example, in tomatoes) and 39.7 μg/kg (in “oats”).^35^ Taking into account a recently described recovery of *Alternaria* toxin mixtures in rat feces/urine up to a total of 15% of orally administered toxins,^21^ exposure scenarios due to a mixed diet within the GIT in the high-nanomolar-to-low-micromolar range are plausible, considering the volumes of single GIT organs to range from 8 to 83 mL, according to Schiller and colleagues.^36^ Of note, due to rare contamination data of ATX-II at this point,^37^ exposure to the perylene quinone compound is likely to occur in lower ranges compared to AOH, which was acknowledged in this study by choosing lower experimental concentrations for ATX-II and a constant ratio of AOH/ATX-II 10:1 in binary mixtures.

### CellTiter-Blue Assay Coupled to Sulforhodamine B (SRB) Assay and CyQuant LDH Assay

For testing cell metabolic activity, cells were seeded in 96-well plates for 48 h. Afterward, the respective incubation solutions were applied for 1, 5, or 24 h. DMSO (1%) and Triton X-100 (0.1%) served as a solvent and a toxic control, respectively. For proinflammatory stimulation, IL-1β (25 ng/mL) was added 2 h into toxin incubation and concomitantly incubated for another 3 or 24 h, respectively. Post incubation, cell monolayers were washed using phosphate-buffered saline (PBS) and a 1:10 dilution of CellTiter-Blue solution in Dulbecco’s modified Eagle medium (DMEM) (21063029, Gibco) was added for 1 h. Fluorescent signals of supernatants were measured utilizing a Synergy H1 hybrid multimode reader (BioTek, Bad Friedrichshall, Germany) at 560 nm_ex_/590 nm_em_.

Subsequently, the cell protein content was investigated by applying the Sulforhodamine B assay (SRB).^38^ Cells were washed once using PBS and fixed by applying 5% TCA solution in dH_2_O for 30 min at 4 °C. Subsequently, wells were washed by applying dH_2_O twice, and the plates were dried overnight at room temperature. Afterward, proteins were stained using 0.4% (w/v) SRB solution in 1% (v/v) acetic acid for 1 h at room temperature in the dark. This was followed by washing steps by applying tap water and 1% acetic acid. Afterward, the plates were dried again overnight. The following day, 10 mM TRIS solution (pH 10; 100 μL per well) was added to the wells, and after 10 min of orbital shaking at 500 rpm, absorbance was measured at 570 nm using a Synergy H1 hybrid multimode reader (Biotek, Bad Friedrichshall, Germany). Furthermore, for selected experimental conditions, the potential to affect cell viability via alteration of the cell membrane/integrity was assessed by applying the CyQuant LDH Cytotoxicity Assay (24 h of incubation) according to the manufacturer’s protocol.

### Quantitative Analysis of Cytokine Gene Transcription

For quantitative real-time PCR (qRT-PCR) experiments to analyze gene transcription levels of the four cytokines IL-8, TNF-α, matrix metallopeptidase 2 (MMP-2), and matrix metallopeptidase 9 (MMP-9), cells were seeded and incubated as described above. Subsequently, cells were harvested, and total RNA was purified utilizing a Maxwell 16 LEV simplyRNA Cells Kit (Promega, Madison, WI) following the manufacturer’s instructions. RNA concentrations were determined via Nanodrop 2000/2000c spectrophotometer (Thermo Fisher Scientific) measurements and stored at −80 °C until further processing. Afterward, RNA was reverse-transcribed into cDNA applying the QuantiTect Reverse Transcription Kit (Qiagen, Hilden, Germany) according to the manufacturer’s instructions and kept at −20 °C. For amplification of DNA fragments of the genes of interest, QuantiTect SYBR Green Master Mix and gene-specific QuantiTect Primer Assays (Qiagen, Hilden, Germany) were applied utilizing a StepOnePlus System (Applied Biosystems). The following primer assays were used: hypoxanthine phosphoribosyltransferase 1 (hPRT1): (Hs_HPRT1_1_SG, QT00059066), hydroxymethylbilane synthase (HMBS): (Hs_HMBS_1_SG, QT00014462), interleukine-8 (IL-8): (Hs_CXCL8_1_SG, QT00000322), tumor necrosis factor α (TNF-α): (Hs_TNF_1_SG, QT00029162), matrix metallopeptidase 2 (MMP-2): (Hs_MMP2_1_SG, QT00088396), and matrix metallopeptidase 9 (MMP-9): (Hs_MMP9_1_SG, QT00040040). A total of 25 ng of cDNA was applied, and DNA amplification was conducted as listed: enzyme activation, 15 min at 95 °C and 40 cycles of 15 s at 94 °C, 30 s at 55 °C, and 3 s at 72 °C. Subsequent melting curve analysis: 15 s at 95 °C and 1 min at 60 °C taken in 0.5 °C steps toward 94 °C for 15 s. Analysis was conducted applying the StepOnePlus software v2.1. Data were normalized to the internal control genes HMBS and HPRT1 and quantified relative to the stimulus control using the 2^–ΔΔCt^ analysis method,^39^ while a similar PCR efficiency was ensured as recommended by Schmittgen and Livak.^40^

### Bio-Plex Multiplex Immunoassay

For the measurement of the secretion levels of proinflammatory cytokines, experimental conditions were chosen based on the gene transcription experiments. Cells were seeded and incubated into 12-well plates according to the procedure for qRT-PCR in three independent experimental setups. After a total of 5 h of incubation, supernatants were removed from cell layers. Supernatants were centrifuged at 10 000*g* and 4 °C for 10 min; thereof, supernatants were collected and stored at −80 °C until further processing. Subsequently, the assay was performed according to the manufacturer’s instructions (Bio-Rad Laboratories, Inc.). Standard solutions and diluted supernatants were briefly incubated with magnetic microparticles coated with the respective antibodies specific to the analytes of interest. Afterward, the particles were incubated with the biotinylated antibody solution and a streptavidin-phycoerythrin conjugate provided within the assay kit. Analysis of samples was conducted in duplicate utilizing a Bio-Rad Bio-Plex 200 System. Calculations were performed implementing a five-parameter logistic (5-PL) curve fit applying Bio-Plex Manager 6.1.

### Immunofluorescence Staining, Image Acquisition, and Analysis

Depending on the experimental layout, cells were incubated with toxins for 1, 5, or 24 h, with or without pre- and concomitant incubation of CH-22 (Supporting Information, Figure S2). Whenever cells were stimulated, 25 ng/mL IL-1β was applied 2 h into toxin incubation. After incubation, cells were fixed by applying a 4% FA solution in PBS, permeabilized using 0.2% Triton X-100 (TX-100) in PBS-A solution, and unspecific binding sites were blocked utilizing 1% bovine serum albumin (BSA) in PBS-A. Primary antibody solutions (1:500, otherwise 1:250 for ZO-1 and Ocln) were prepared in 0.25% BSA and applied for 2 h of incubation. Primary antibodies were removed using 0.05% TX-100 in PBS-A (washing buffer). Subsequently, species-specific secondary antibodies (dil. 1:1000) were incubated for 1.5 h. After two washing steps (washing buffer), antibodies were fixed by applying 4% FA solution. Subsequently, a quenching solution was applied (0.75% glycine solution in PBS-A). Afterward, slides were mounted using ROTI-Mount FluorCare DAPI and kept at 4 °C until imaging. Acquisition of images for tight junction and adherens junction membrane protein staining was conducted using an LSM Zeiss 710 microscope equipped with an ELYRA PS.1 system, an AndoriXon 897 (EMCCD) camera, and a Plan Apochromat 63X and 100X (1.46 NA) objective. Image analysis of regions of interest (ROIs) was performed in Fiji.^41^ Signal intensities were obtained using the calculated corrected total cell fluorescence (CTCF): CTCF = Integrated Density – (Area of selected ROI × mean fluorescence of background selection). A minimum of three individually conducted experiments was performed, each resulting in at least seven randomly chosen optical fields per incubation condition, leading to a minimum of 35 analyzed cells/data points (Figure 8). For high-density monolayer immunofluorescence imaging experiments (Figures 9 and 10), a minimum of four individually conducted experiments were performed; each biological replicate was imaged in at least three technical replicates, and at least three randomly chosen cells were analyzed thereof, respectively. The perinuclear area was defined as distance *P* from the center of the nucleus (*n*) in nanometer: distance *P* = Ø*_n_*/2. The cytosolic area was defined as distance *C*: distance *C* = Ø*_n_*/4 + distance *P*. Image acquisition for all other immunofluorescence staining experiments was performed utilizing a Lionheart FX Automated Microscope (Biotek Instruments, Inc., Winooski, VT) and its analysis, via the integrated Image Analysis Software Gen 5.08. Immunofluorescence staining experiments were performed in at least three individual setups, each including at least two technical replicates per incubation condition. Automated image acquisition was conducted by applying a 20× objective and focusing on four to nine different optical fields per technical replicate, resulting in a minimum of 24 different optical fields/data points, for which cytosolic/nuclear immunofluorescence of single cells was analyzed (Figures 4–7). For analysis of nuclear translocation of the selected targets, Gen 5.08 (BioTek) software was set up to apply a fixed threshold against the background and a primary mask was set for the stained nuclei of the cells with a secondary mask representing the cell border. Automated calculation of a translocation ratio was set up using corrected fluorescence values of nucleus/cytosol. Oversaturated or blurry optical fields were excluded from the analysis.

### Experimental Setup and Statistical Analysis

All experiments conducted were performed in at least three independent experiments and respective technical replicates as stated in the Results section. Compiled data points for all experiments were normalized to the corresponding solvent control (or IL-1β stimulus control, if applicable). Data sets were checked for normality (Shapiro–Wilk, *p* < 0.05), and a Nalimov Outlier test was applied prior to statistical testing using the software Origin Pro 2020. Details on individually applied parameters and types of tests can be found in the respective figure captions.

## Results

### *Alternaria* Toxins Suppress mRNA Levels of Proinflammatory Cytokines and Impact mRNA Levels of Matrix Metallopeptidases

**Figure 2:**
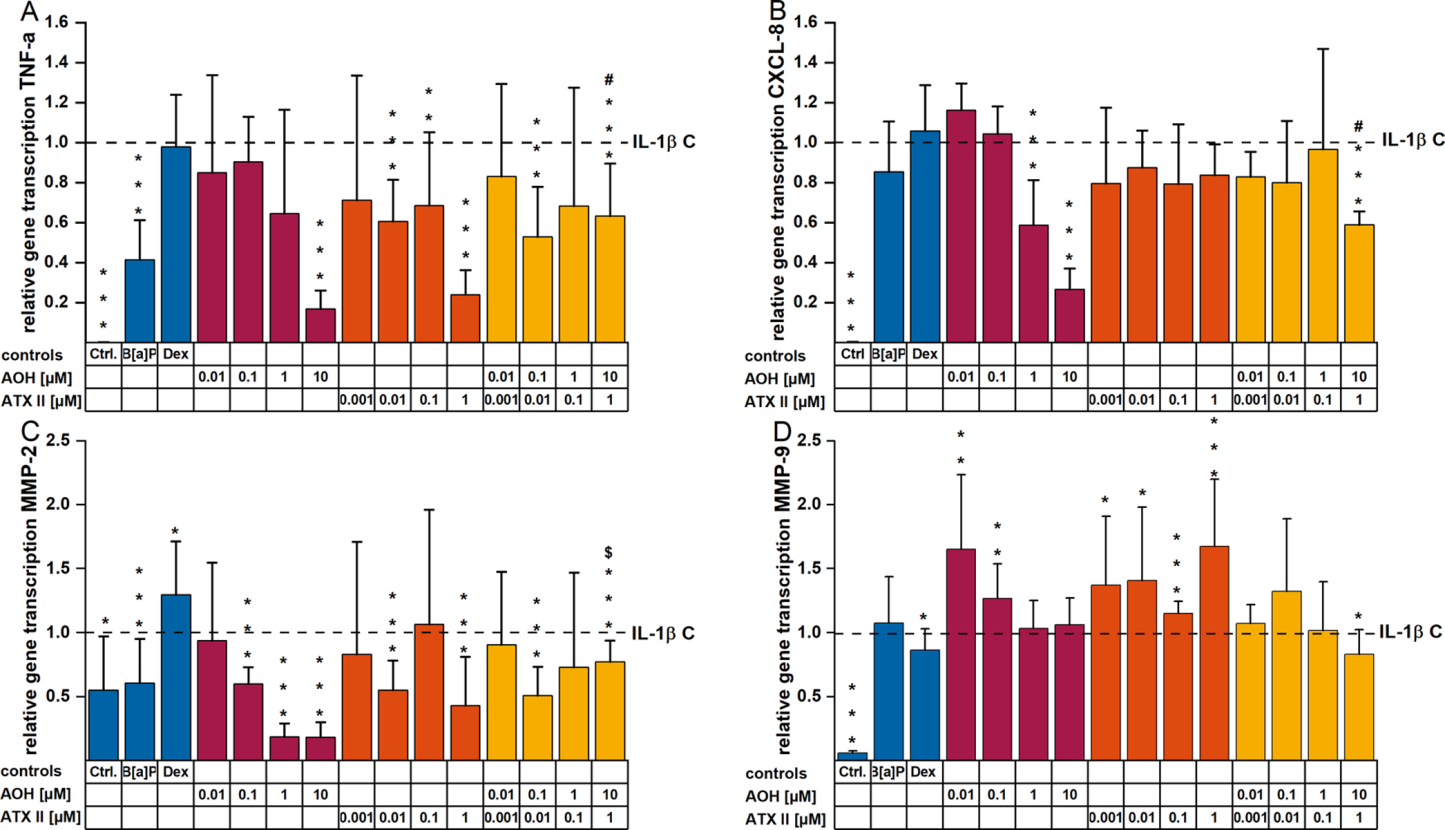
Effects of AOH, ATX-II, and a mixture of both on relative gene transcription levels of (A) *TNF-a* (encoding for TNF-α), (B) *CXCL*-8 (encoding for IL-8), (C) *MMP*-2, and (D) *MMP*-9 in IL-1β-stimulated HCEC-1CT cells measured with qRT-PCR; mRNA levels are normalized to the endogenous controls HPRT1 and HMBS and are presented as relative quantity (RQ) in comparison to solvent control-treated with IL-1β (25 ng/mL) for 3 h, which was set to 1. Cells were treated with respective compounds for 5 h, while after 2 h of incubation, IL-1β was added as an inflammatory stimulus. Data are expressed as mean values + SD normalized to IL-1β C (control) and represent three to seven individually conducted experiments. Significant differences to IL-1β stimulation were calculated applying a two-sample *t*-test and are indicated with * (*p* < 0.05), ** (*p* < 0.01), and *** (*p* < 0.001). Combinatory effects marked with # were calculated to be significantly different from both single compounds’ effects; $ indicates significant differences against the effect of AOH alone (*p* < 0.05).

The potential of *Alternaria* toxins to modulate the mRNA expression level of the proinflammatory cytokines TNF-α and IL-8 triggered upon inflammatory stimulation was evaluated by qRT-PCR. The incubation condition applying 25 ng/mL IL-1β for 3 h was used to induce cytokine mRNA levels and set as a positive control. Both *Alternaria* toxins could suppress the elevation of the respective cytokine mRNA level relative to the inflammatory stimulus: this effect was visible when cells were preincubated with AOH or ATX-II singularly or in combination for 2 h prior to IL-1β stimulation. A suppressive effect of the cytokine TNF-α mRNA was observed for AOH in a concentration-dependent manner starting at 1 μM. Significant suppression was obtained after incubation with 10 μM AOH, which reduced cytokine transcription to 16.8 ± 0.09 in comparison to the IL-1β stimulated controls (Figure 2A). For ATX-II, a reduction was evident for all concentrations tested, with the highest impact induced by 1 μM (reducing the level of IL-1β-induced TNF-α mRNA to 0.239 ± 0.12 relative gene expression). The binary mixture AOH/ATX-II (10:1) was less effective in the modulation of TNF-α transcript levels, while still exerting a significant suppressive effect. The anti-inflammatory corticosteroid Dex did not impact the TNF-α transcript levels in our cell system. The positive control for AhR activation, 1 μM B[*a*]P significantly suppressed TNF-α expression compared to the IL-1β control (Figure 2A). Concerning IL-8 expression levels, this was reduced in a concentration-dependent manner (Figure 2B). Suppressions were observed starting from 1 μM AOH and exhibited the highest potency at 10 μM (0.266 ± 0.105-fold) toward gene expression. Diverging from TNF-α results, incubation with ATX-II had a minor impact on IL-8 gene expression levels compared to the IL-1β stimulated positive control. The combined exposure to AOH/ATX-II (ratio 10:1) showed no impact on IL-8 transcript levels, except for the highest concentrations (10 μM AOH and 1 μM ATX-II), which exhibited significant suppression. B[*a*]P 1 μM caused no measurable changes in IL-8 mRNA levels.

To determine the potential impact of the *Alternaria* toxins on inflammation-induced alterations of the mRNA expression of the genes for the two matrix metallopeptidases MMP-2 and MMP-9, qRT-PCR experiments were conducted applying the same conditions as for the proinflammatory cytokines. Thanks to 3 h of IL-1β stimulation, transcription levels of both enzymes were increased (Figure 2C,D). For AOH, dose-dependent suppression of MMP-2 mRNA was observed: in this case, the most potent suppression was achieved by 1 μM and 10 μM AOH (diminishing gene transcription to 0.18 ± 0.107 and 0.18 ± 0.118, respectively; Figure 2C). The complete concentration range of ATX-II led to decreased MMP-2 mRNA expression levels compared to the IL-1β control, with 1 μM showing the most extensive impact reducing mRNA levels 0.43 ± 0.383-fold. The combined application of AOH and ATX-II (ratio 10:1), while still exerting slight suppressive effects at some concentrations on the metalloprotease transcription, was less potent compared to the toxins applied singularly. Dex slightly enhanced MMP-2 transcription levels, while 1 μM B[*a*]P led to a moderate, yet significant suppression of MMP-2 mRNA levels (Figure 2C). A different pattern was found for MMP-9 mRNA gene transcription (Figure 2D). Stimulation with IL-1β resulted in induced transcription levels that were only marginally altered by preincubation with any test compound. AOH (0.01 and 0.1 μM) and ATX-II (all concentrations) further enhanced MMP-9 as single toxins; however, this effect vanished in the combinatory exposure scenario.

### *A. alternata* Toxins Suppress IL-8 Secretion in IL-1β Stimulated HCEC-1CT Cells

**Figure 3:**
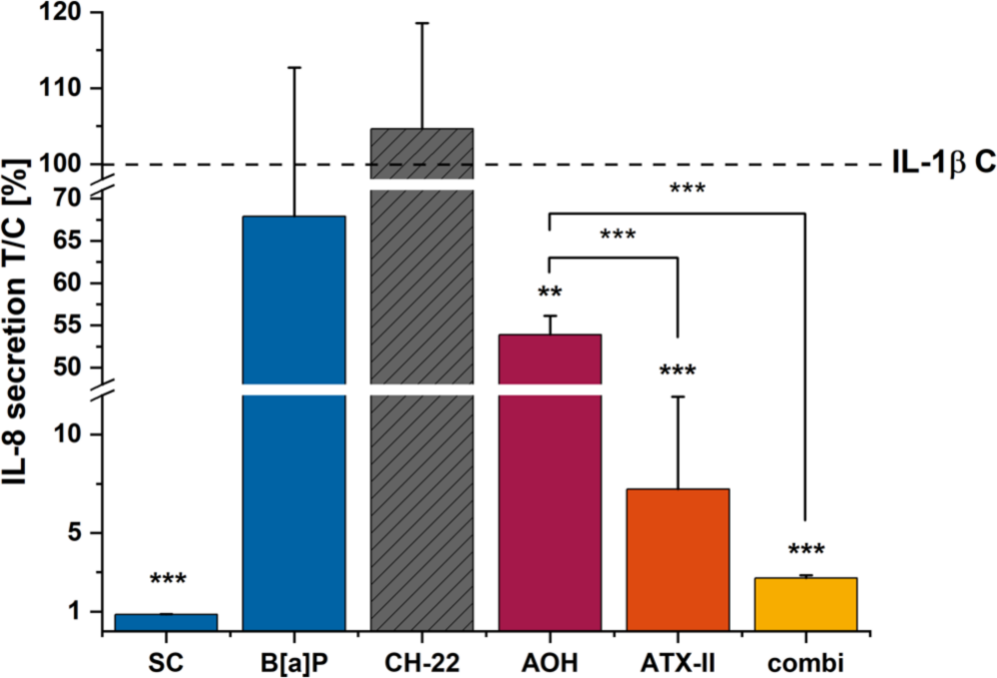
IL-8 secretion levels after 5 h of incubation (3 h + IL-1β) determined via the Bio-Plex Luminex Assay. Protein secretion levels were determined quantitatively and normalized to the stimulus control. For significant differences compared to the IL-1β control, the Student’s *t*-test was applied for *p* < 0.01 ** and *p* < 0.001 ***. Incubation conditions include B[*a*]P 1 μM, CH-22 1 μM, AOH 10 μM, ATX-II 1 μM, and binary mixture AOH/ATX-II (10:1) (combi).

Cytokine secretion was evaluated by applying a Luminex Bio-Plex Assay to determine whether the changes in transcription levels of the proinflammatory cytokine IL-8 would be reflected at the protein secretion level. IL-1β stimulation enhanced the secretion levels of IL-8 117-fold compared to the basal level of the solvent control (SC: 71.3 pg/mL; IL-1β C: 8404.4 pg/mL; Figure 3). An agonist of the AhR receptor, B[*a*]P,^42^ and the antagonist CH-22^33^ (both 1 μM) were included to investigate the involvement of this pathway in the cytokine secretion at the intestinal level. B[*a*]P slightly dampened the IL-8 induction to 7505.6 pg/mL (67.9% compared to IL-1β), while CH-22 1 μM had no effect (8796.4 pg/mL; 104.7%). Both *Alternaria* toxins as single compounds and in combination suppressed IL-8 secretion. AOH reduced IL-8 secretion to 4528.9 pg/mL (53.9%), ATX-II even further to 607.3 pg/mL (7.2%), and the binary mixture suppressed IL-8 secretion almost completely, resulting in 226.9 pg/mL (2.7%).

### *Alternaria* Toxins Trigger the Translocation of the Transcription Factors AhR and Nrf2

**Figure 4:**
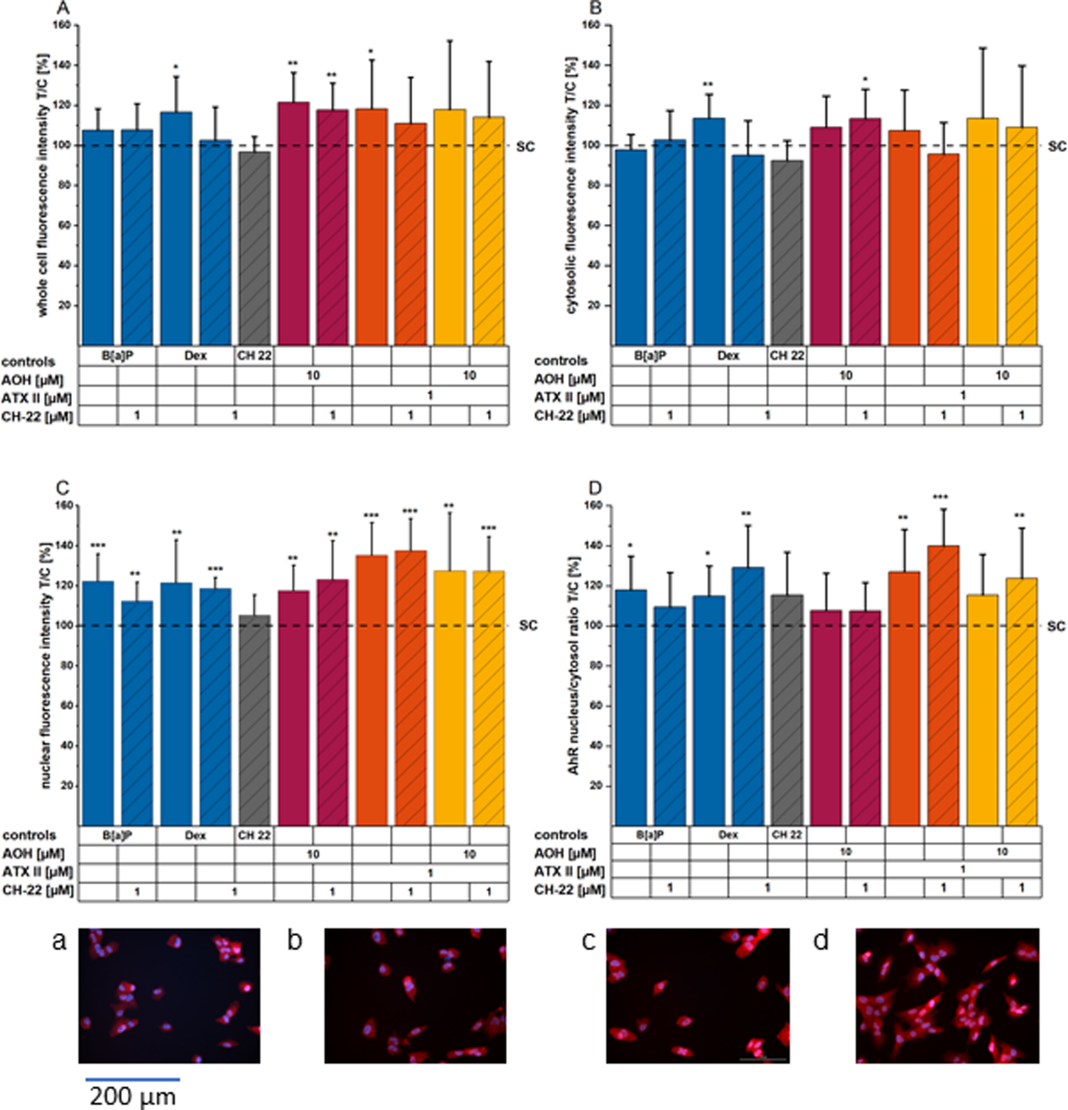
Immunofluorescence imaging of AhR (A–D) translocation after 1 hour of incubation. AhR fluorescence signals throughout the whole cells, (A) within the cytosolic (B) and nuclear (C) fractions, and the calculated ratio (nucleus/cytosol, D). Images showing immunofluorescence stainings of (HCEC-1CT). Images (a–d) show AhR (red) and nuclei (blue) of HCEC for the following conditions: (a) solvent control, (b) CH-22 1 μM, (c) B[*a*]P 1 μM, and (d) ATX-II 1 μM. Bar graphs depict AhR immunofluorescence signals within the respective cell compartment or as a nucleus/cytosol ratio (D, H) presented as mean + standard deviation (SD) of at least four individual experiments conducted in a minimum of technical duplicates with at least four optical fields per technical replicate. The data are normalized to the ratio of the solvent control, and significances were calculated applying a two-sample *t*-test at *p* < 0.05: *, *p* < 0.01: **, and *p* < 0.001: ***; asterisks indicate significant differences compared to the solvent control, and # indicates significant differences of the combinations of a compound and CH-22 compared to the compound alone. All images were taken using a 20× objective; therefore, the indicated scale bar applies to all images depicted.

**Figure 5:**
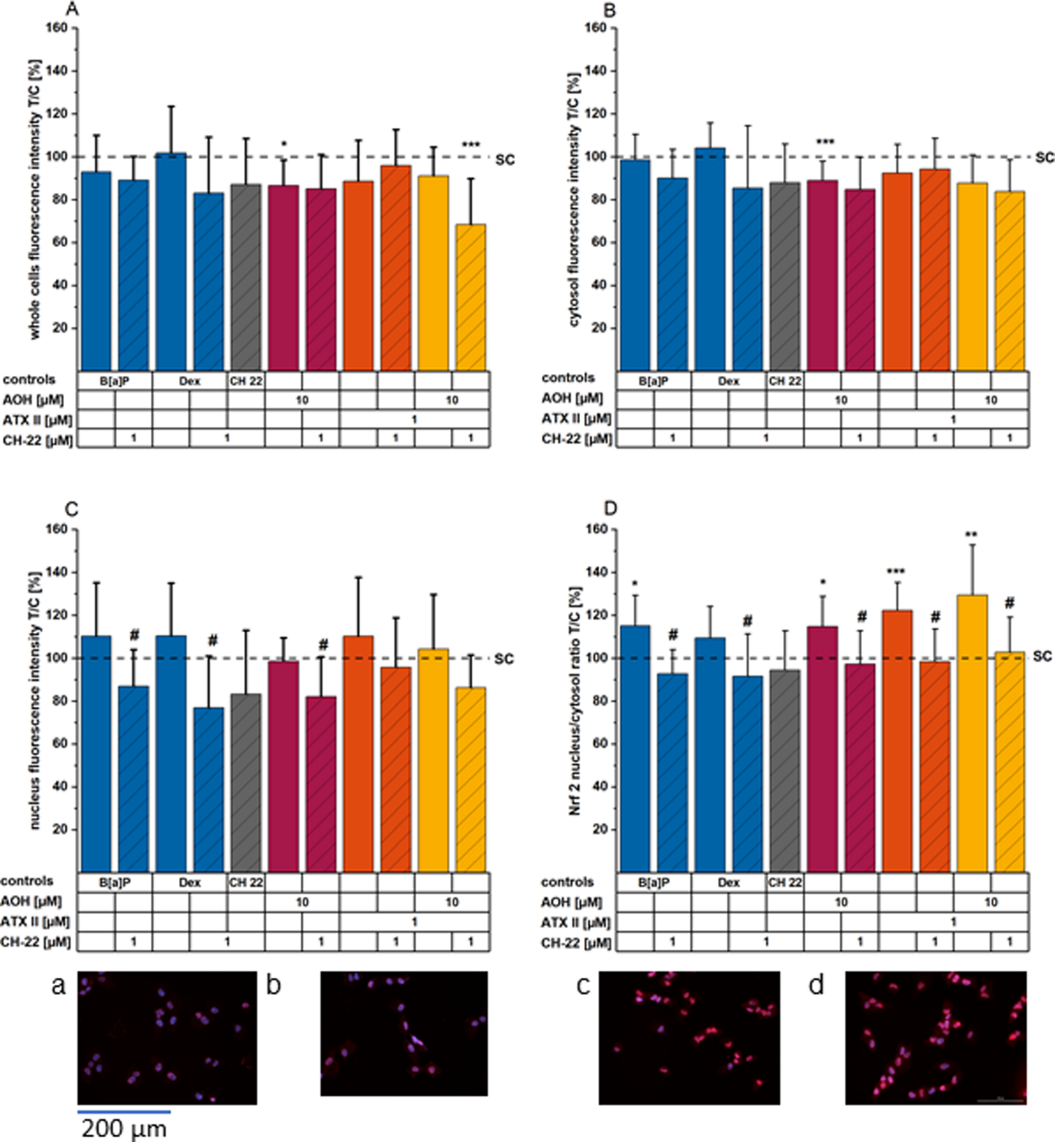
Immunofluorescence imaging of Nrf2 (A–D) translocation after 1 hour of incubation. Bar graphs show Nrf2 fluorescence signals throughout the (A) whole cells, within the cytosolic (B) and nuclear (C) fraction, and the calculated ratio (nucleus/cytosol, D). Images showing immunofluorescence stainings of HCEC-1CT. Images (a–d) show Nrf2 (red) and nuclei (blue) of HCEC for the following conditions: (a) solvent control, (b) CH-22 1 μM, (c) B[*a*]P 1 μM, and (d) ATX-II 1 μM. Bar graphs depict Nrf2 immunofluorescence signals within the respective cell compartment or as the nucleus/cytosol ratio (D) presented as mean + SD of at least four individual experiments conducted in a minimum of technical duplicates with at least four optical fields per technical replicate. The data are normalized to the ratio of the solvent control, and significances were calculated applying a two-sample *t*-test at *p* < 0.05: *, *p* < 0.01: **, and *p* < 0.001: ***; asterisks indicate significant differences compared to the solvent control, and # marks significant differences of the combinations of a compound and CH-22 compared to the compound alone. All images were taken using a 20× objective; the indicated scale bar applies to all images depicted.

To investigate the potential effects of the toxins on the AhR and Nrf2/ARE pathway in HCEC-1CT, immunofluorescence experiments were performed to obtain information on the nuclear translocation capacity of AhR and Nrf2. A robust AhR translocation could be observed after incubation with 1 μM ATX-II (Figure 4D), which was similar to the positive control B[*a*]P 1 μM. Of note, the reportedly full antagonist CH-22^33^ only decreased AhR translocation induced by B[*a*]P but not by ATX-II 1 μM. Essentially for the evaluation, ATX-II and B[*a*]P significantly enhanced the immunofluorescence signal of AhR, specifically in the nuclear region. Besides the immunolocalization being elevated in the whole cell, calculation of the nucleus/cytosolic signal ratio confirmed this behavior, suggesting effective translocation. In this respect, the response triggered by AOH followed a different pattern. After 1 h of incubation with the mycotoxin, fluorescence localization of AhR was significantly enhanced in the nucleus and within the whole cell, suggesting AOH to rather increase overall AhR mobilization. The same trend was observable for the binary mixture (Figure 4A–D). For Nrf2, all substances incubated singularly, as well as the 10:1 combination of AOH and ATX-II, significantly altered the cellular distribution of the transcription factor (Figure 5A–D, Dex not significant). While single and combinatory toxin incubations led to none or slight enhancements of fluorescence signals within the nucleus (ATX-II in particular), reductions in overall detection for the whole cell and/or the cytosolic compartment were found for all tested conditions (significantly for AOH). In sum, the nucleus/cytosolic signal ratio was enhanced to increasing extents in this order: AOH 10 μM – ATX-II 1 μM – AOH + ATX-II binary mixture (Figure 5D). Notably, pre- and concomitant incubation of CH-22 resulted in suppression of enhanced translocation. As previously described for AhR, CH-22 alone did not alter the Nrf2 distribution within the cells, when compared to the solvent control.

### *Alternaria* Toxins Activate AhR under Inflammatory Conditions and Modulate the Translocation of the NF-κB/p65 Transcription Factor

**Figure 6:**
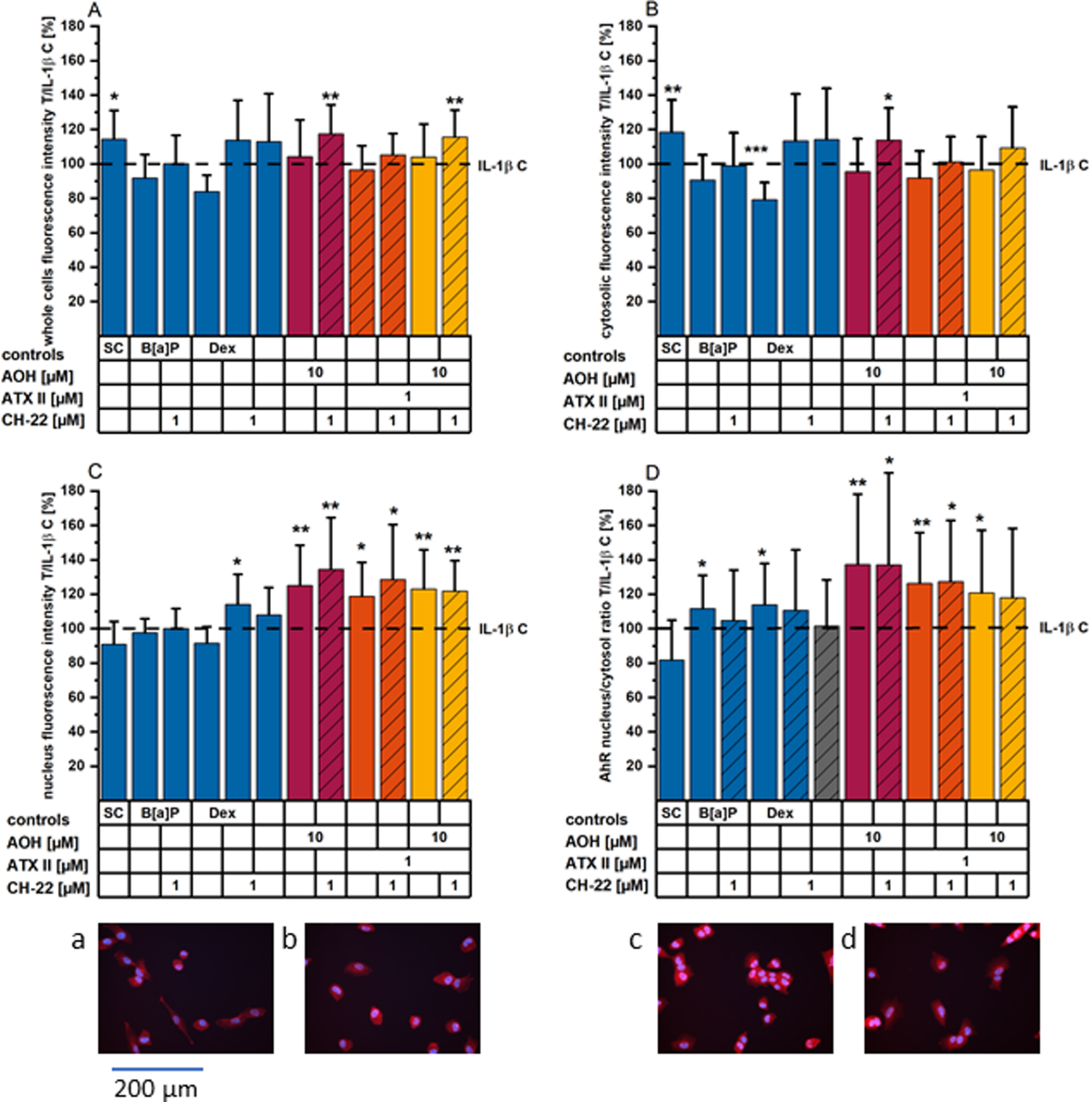
AhR translocation experiments after 5 hours of incubation (+3 hours pro-inflammatory stimulation). Bar graphs show AhR immunofluorescence as T/IL-1β C for (A) whole cells, (B) the cytosolic, and (C) nuclear fraction, and (D) the ratio of nucleus/cytosol. Images (a–d) show AhR (red) and nuclei (blue), images (e–h) show NF-κB p65 (red) and nuclei (blue) of HCEC when exposed to the following conditions: (a) solvent control, (b) 25 ng/mL IL-1β, (c) 1 μM B[*a*]P, and (d) 10 μM AOH for a total of 5 h (toxins). Bar graphs depict immunofluorescence data of AhR (A–D) presented as means + SD normalized to the IL-1β control of a minimum of four individual experiments conducted, including at least technical duplicates in each and four optical fields. Significant differences compared to the stimulus control were calculated applying a two-sample *t*-test and are highlighted as the following: * (*p* < 0.05), ** (*p* < 0.01), and *** (*p* < 0.001). # represents significant differences compared to the solvent control (at *p* < 0.05). All images were taken using a 20× objective; therefore, the indicated scale bar applies to all images depicted.

**Figure 7:**
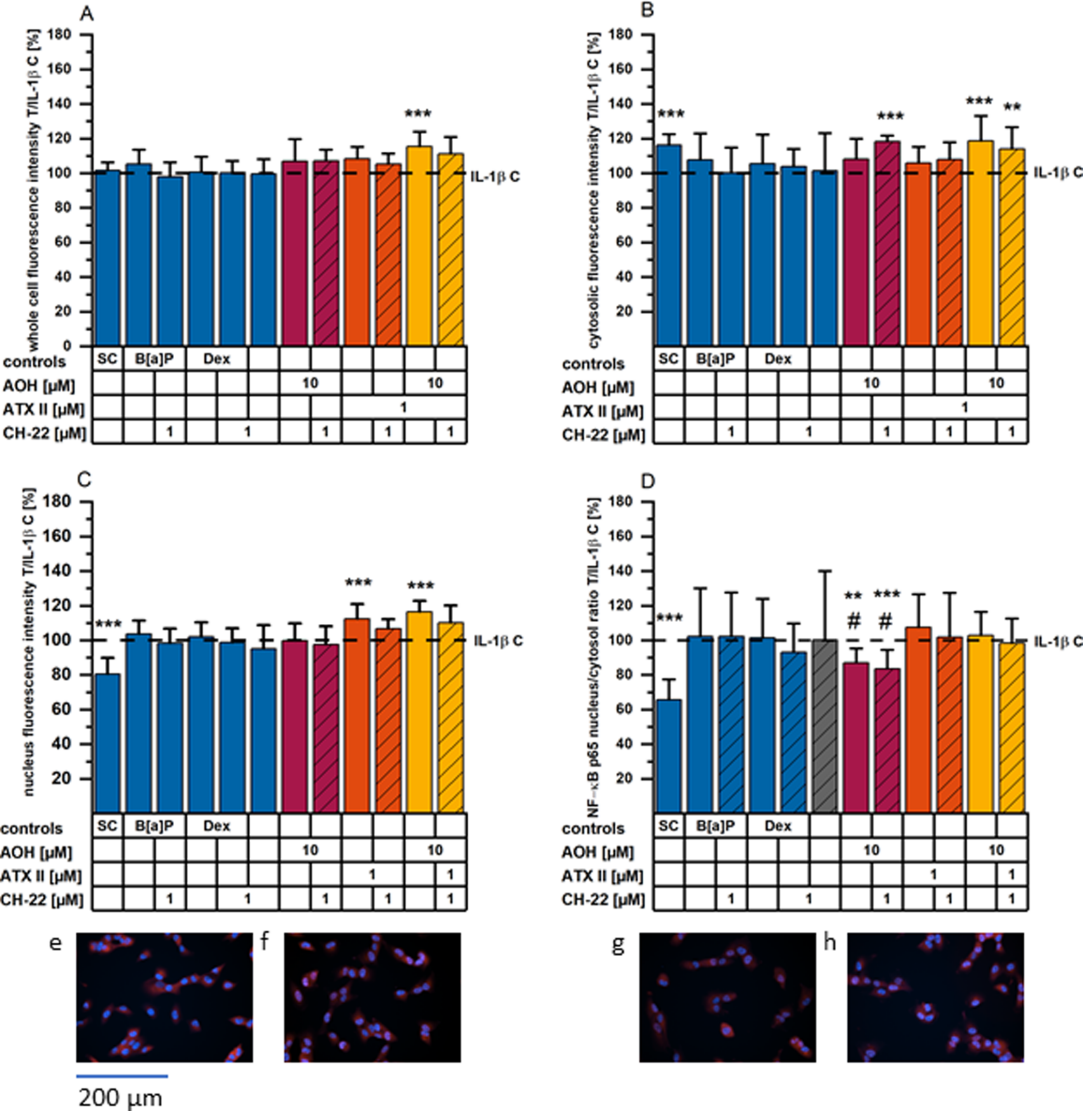
NF-kB/p65 translocation experiments after 5 hours of incubation (+3 hours of pro-inflammatory stimulation). Bar graphs show p65 immunofluorescence as T/IL-1β C for (A) whole cells, (B) the cytosolic, and (C) nuclear fraction, and (D) the ratio of nucleus/cytosol. Images (e–h) show NF-κB p65 (red) and nuclei (blue) of HCEC when exposed to the following conditions: (a) solvent control, (b) 25 ng/mL IL-1β, (c) 1 μM B[*a*]P, and (d) 10 μM AOH for a total of 5 h (toxins). Bar graphs depict immunofluorescence data of the NF-κB p65 (A–D) presented as means + SD normalized to the IL-1β control of a minimum of four individual experiments conducted, including at least technical duplicates each and four optical fields. Significant differences compared to the stimulus control were calculated applying a two-sample *t*-test and are highlighted as the following: * (*p* < 0.05), ** (*p* < 0.01), and *** (*p* < 0.001). # represents significant differences compared to the solvent control (at *p* < 0.05). All images were taken using a 20× objective; therefore, the indicated scale bar applies to all images depicted.

To assess possible interactions of AhR activation and NF-κB pathway activation, translocation of the AhR and the NF-κB/p65 protein was also investigated via immunofluorescence experiments. As for qRT-PCR experiments, inflammatory stimulation was provided with IL-1β incubation starting 2 h into toxin exposure (total incubation time: 5 h). Proinflammatory stimulation moderately, yet not significantly, enhanced the translocation of AhR into the nucleus (Figure 6A–D). Nonetheless, all compounds incubated alone, including B[*a*]P and Dex, as well as the combinatory mixture of 10 μM AOH and 1 μM ATX-II, led to a significant nuclear translocation of AhR. Following the results of the AhR/Nrf2 immunofluorescence, 1 μM CH-22 pre- and concomitantly incubated with B[*a*]P reduced the AhR translocation induced by B[*a*]P alone, while it could not suppress the translocation of the receptor induced by the other treatments nor modify the activation profile *per se*. In line with a proinflammatory response, IL-1β triggered NF-κB/p65 translocation into the nucleus; however, the majority of the treatments applied prior to IL-1β showed no modulatory effect in this respect (Figure 7A–D). However, AOH 10 μM successfully reduced nuclear translocation of NF-κB/p65. Of note, CH-22 pre- and concomitant incubation with AOH and further IL-1β inhibited a vast nuclear translocation of p65 protein as well. Nonetheless, in this experimental layout, AOH alone and in combination with CH-22 were not sufficient to restore the p65 translocation to the ratio observed for the solvent control.

### *Alternaria* Toxins Enhance Tight Junction and Adherens Junction Membrane Proteins Cldn 4, ZO-1, and Integrin β1 in the Inflamed Environment

**Figure 8:**
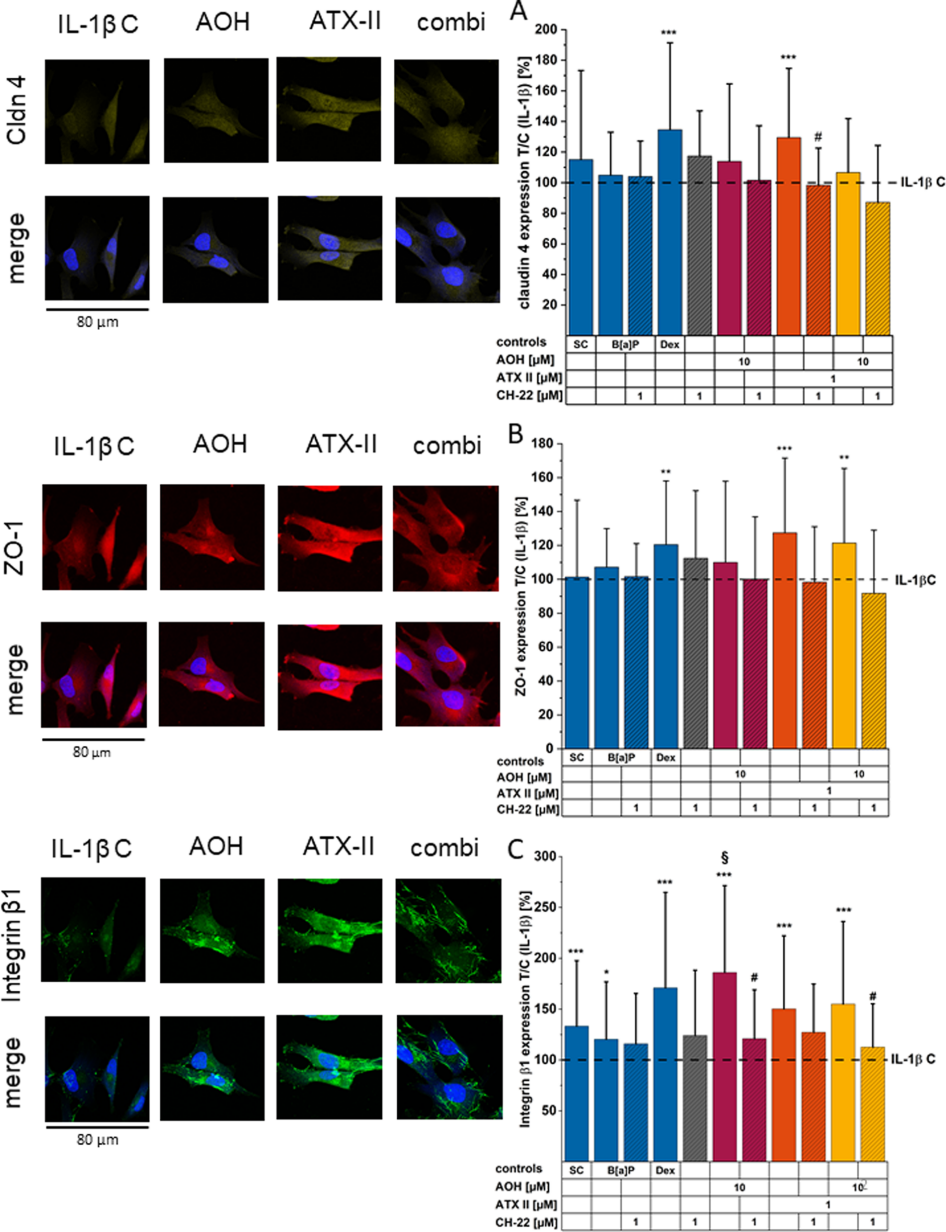
Transmembrane protein expression levels. (A) Cldn 4, (B) ZO-1, and (C) integrin β1 expression was measured after 24 h of toxin exposure to HCEC-1CT. Images show Cldn 4, ZO-1, and integrin β1 expression for the following incubation conditions: IL-1β, 10 μM AOH, 1 μM ATX-II, and the binary mixture (combi). Corrected cell fluorescence intensities (CTCFs) (bar graphs) were normalized to the IL-1β stimulus control and are expressed as means + SD of a minimum of three independent experiments, including at least six randomly chosen optical fields per condition. Significant differences were calculated by applying a two-sample *t*-test. Differences of incubation conditions compared to the IL-1β control are indicated by *(*p* < 0.05), ** (*p* < 0.01), and *** (*p* < 0.001), differences between incubation of a compound + CH-22 and the compound alone are highlighted with # (*p* < 0.05), and significant differences compared to solvent control are shown as § (*p* < 0.05).

Since barrier function relies on cell morphology, we tested whether *Alternaria* toxins could impact membrane proteins involved in the maintenance of structural tissue homeostasis. Particularly, we focused on members of protein families already described for their connection with AhR, NF-κB, or Nrf2 pathways such as Cldn 4, ZO-1, and integrin β1.^15,16,43^ After 24 h of incubation with IL-1β, inflammatory stimulation slightly reduced Cldn 4 expression (Figure 8A); this effect was counteracted by the anti-inflammatory Dex (1 μM). The control compounds for the AhR pathway, B[*a*]P (1 μM), and CH-22 (1 μM) exerted no obvious alteration in Cldn 4 expression. The toxin AOH could only slightly, yet not significantly, enhance the immunofluorescence signal of Cldn 4. However, ATX-II (1 μM) significantly increased the protein localization. This effect was counteracted by the co-incubation with CH-22. Remarkably, the binary toxin mixture led to no obvious alteration in Cldn 4 expression (Figure 8A). ZO-1 expression (Figure 8B) was not altered by inflammatory stimulation alone; yet in this case, Dex triggered a significant increase in the ZO-1 immunofluorescence signal. Among the treatments, ATX-II triggered a significant enhancement of ZO-1 immunofluorescence in the cytosol, which could again be diminished by co-incubation with CH-22. Intriguingly, the expression level of this tight junction protein was elevated by the binary toxin mixture (AOH/ATX-II 10:1), and this change was successfully prevented by pre- and co-incubation with CH-22 (Figure 8B). Cytosolic immunolocalization of integrin β1 (Figure 8C) was significantly suppressed by IL-1β cytokine stimulation. This effect was counteracted by incubation with all control conditions, except CH-22. Further, 10 μM AOH significantly enhanced the cytosolic signal of integrin β1, exceeding the level of the solvent control significantly. ATX-II and the binary toxin mixture likewise elevated the immunofluorescence signal, even though not quite as much. Intriguingly, prior and co-incubation with CH-22 suppressed the toxins’ effects alone (for AOH) and in the binary incubation condition significantly (Figure 8C).

### Altertoxin II Alters Colonic Cell Morphology and ZO-1 Distribution and Enhances Ocln Protein Expression under Noninflammatory and Inflamed Conditions

**Figure 9:**
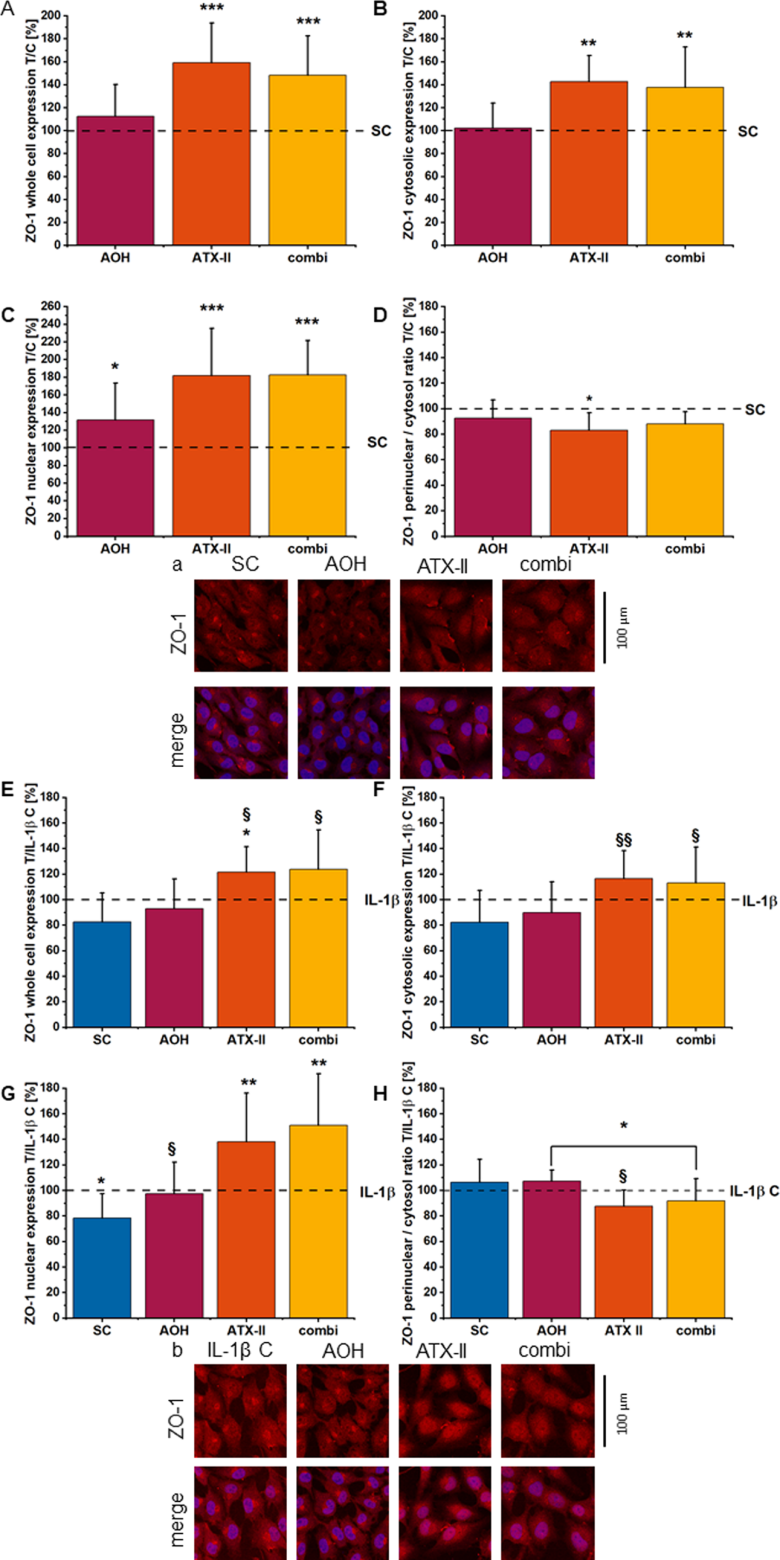
Immunofluorescence staining of ZO-1 on a high-density monolayer of HCEC-1CT incubated for 24 h with toxins alone (AOH 10 μM, ATX-II 1 μM, combi = binary mixture) or concomitantly with IL-1β stimulus. ZO-1 distribution between the perinuclear and cytosolic regions was calculated as a ratio and normalized to the respective control conditions (A + B). (C + D) panels show Ocln staining for the respective conditions. Mean values of CTCF for chosen ROIs of at least four biological replicates and technical triplicates were normalized to the controls. Significant differences between the respective controls were calculated by applying the Student’s *t*-test at *p* < 0.05 and *p* < 0.001. § (*p* < 0.05) and §§ (*p* < 0.01) mark significant differences of IL-1β stimulated exposure conditions against the solvent control (no stimulus).

**Figure 10:**
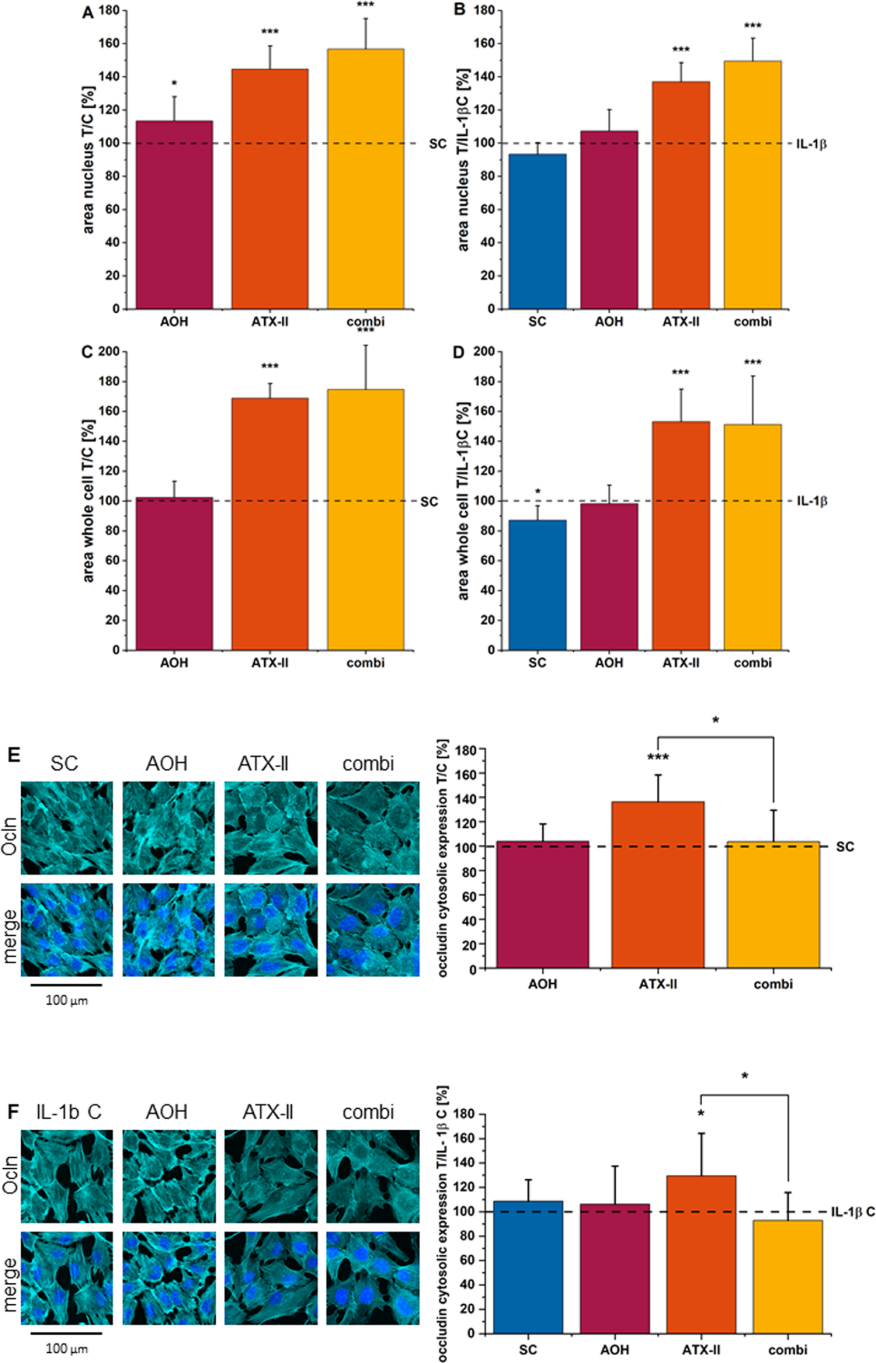
Nuclear and whole-cell area of HCEC-1CT cells incubated for 24 h with 10 μM AOH, 1 μM ATX-II, and the binary mixture (=combi) without stimulus (A + C) and 24 h with 22 h of concomitant IL-1β incubation (B + D). Mean areas of at least four biological replicates and technical triplicates were measured in nm^2^ and normalized to the solvent control or stimulus control, respectively. Panels (E + F) show immunofluorescence staining of Ocln protein expression. Mean CTCF values of at least four biological replicates and technical triplicates were normalized to the respective control conditions. Significant differences to the control conditions were calculated by applying the Student’s *t*-test at *p* < 0.05 * and *p* < 0.001 ***.

To examine the *Alternaria* toxins’ potential to influence tight junction protein expression and distribution, immunofluorescence staining of ZO-1 and Ocln was conducted on a high-density cell monolayer after 24 h of exposure. ZO-1 localization was evaluated for the nuclear, perinuclear, and cytosolic regions, as defined in the Methods section. Upon incubation with the mycotoxins, significant changes could be observed for ZO-1 distribution throughout the cells even in noninflamed conditions. Whole-cell localization levels of ZO-1 were significantly enhanced due to exposure to ATX-II 1 μM and the binary mixture of AOH/ATX-II (10:1, Figure 9A). Signal intensity presented the highest intensity in the nuclear region and at the cell periphery in the cytosolic area, with only a slight reduction for ATX-II in the intermediate/perinuclear segment (Figure 9B–D). For AOH 10 μM treatments, the accumulation of ZO-1 seemed localized in the central part of the cells in correspondence to the nucleus (Figure 9A). In the case of inflammation, IL-1β slightly enhanced the overall expression of ZO-1 protein within all compartments, albeit being significant only in the central region (Figure 9E–H). In these experimental settings, ATX-II and AOH/ATX-II (10:1) were still effective in increasing the localization/recruitment of ZO-1 (Figure 9E–H). Of note, incubation with the toxins significantly modified HCEC morphology, with slight differences in the presence or absence of the inflammatory stimulus (Figure 10A–D). Exposure to AOH or ATX-II as single compounds increased the nuclear area, and the effect on the morphometric descriptor was further increased by the binary mixture (Figure 10A). Retracing the changes in the signal distribution of ZO-1, ATX-II alone, and in the binary mixture significantly increased the cell area spread (Figure 10C). These effects were persistent even in the presence of IL-1β (Figure 10B,D).

In addition to the effects on cell morphology, ATX-II 1 μM significantly enhanced the signal of the tight junction protein Ocln expression. AOH and the binary mixture had no effect on Ocln (cytosolic compartment fluorescence) (Figure 10E). These effects were reproducible also in the presence of IL-1β (Figure 10F).

### Cell Metabolic Activity and Cell Viability Were Marginally Impacted by *Alternaria* Toxins

**Figure 11:**
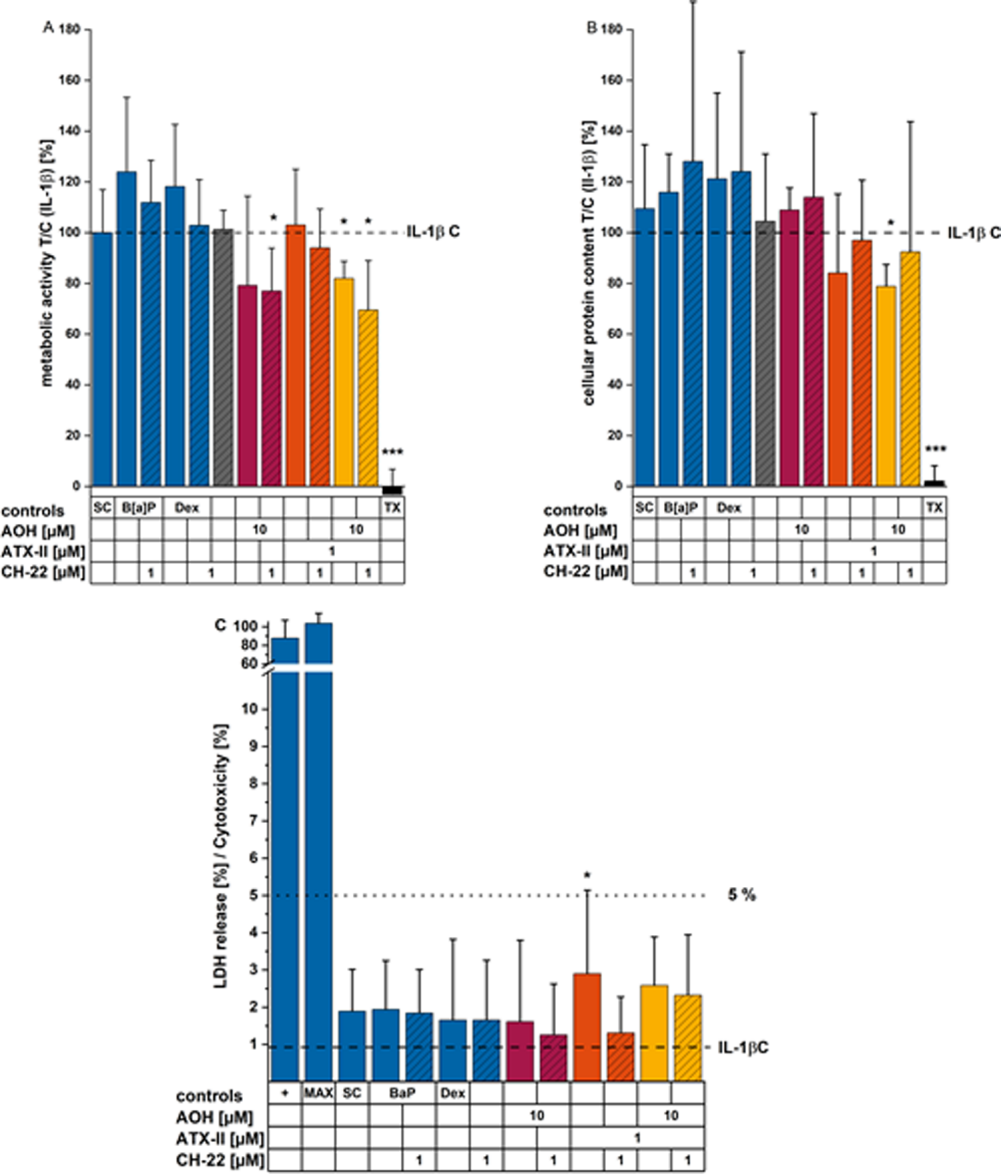
Impact of *Alternaria* toxins on the viability of HCEC-1CT cells. Viability was assessed by (A) metabolic activity (CTB assay), (B) protein content (SRB assay), and (C) LDH release after 24 h of incubation with concomitant IL-1β stimulation for the final 22 h. Results are presented as mean + SD normalized to stimulus control (1% DMSO and 25 ng/mL IL-1 β). “*” (*p* < 0.05) indicates significant differences calculated by applying the Student’s *t*-test test against the stimulus control. “+” and “MAX” indicate the positive control and Lysis control, respectively, provided with the CyQuant LDH Cytotoxicity Kit.

To verify the viability of the cells under our experimental conditions, CellTiter-Blue (CTB) assay was conducted after 5 and 24 h of incubation (Figure 11). Within 3 h, the inflammatory stimulation by IL-1β did not alter the cells’ metabolic activity; *Alternaria* toxins singularly and in the binary mixtures (including the respective combinations with 1 μM CH-22) reduced cell metabolic activity after 5 h. Of note, all of these reductions still resulted in more than 80% metabolic activity throughout all concentrations (Supporting Information Figure S1). At a longer incubation time (24 h), the metabolic activity of HCEC-1CT colon cells was reduced significantly for the following conditions: 10 μM AOH + 1 μM CH-22, 10 μM AOH + 1 μM ATX-II as a binary mixture, and in combination with 1 μM CH-22 (Figure 11A). However, protein content measurements (SRB assay) supported only minor cytotoxic effects in HCEC-1CT after 24 h of exposure to both toxins in the binary mixture, yet not exceeding a loss in cell viability of 21% (remaining protein content after ATX-II + CH-22 incubation: 79 ± 23% in comparison to the IL-1β control, Figure 11B). After 24 h of exposure, the LDH release assay was also conducted to check potential effects on cell membrane integrity. None of the conditions tested showed clear lytic potential, as LDH release never exceeds 5%. The highest increase in LDH release could be observed for ATX-II at 1 μM (2.9 ± 2.2%) (Figure 11C).

## Discussion

In the present study, we explored the immunomodulatory potential of *A. alternata* toxins in human nontransformed epithelial colon cells HCEC-1CT. In addition, we aimed at deciphering the toxins’ impact on colonic tissue homeostasis and epithelial barrier function. Our data suggest the two *A. alternata* toxins AOH and ATX-II to exert immunosuppressive potential as single compounds and in binary combination on nontransformed colonic epithelial cells. In this context, our results indicate the involvement of the AhR, Nrf2 pathway, and their interactions with the proinflammatory NF-κB signaling cascade. Moreover, we described the capability of the two toxins to affect membrane-bound proteins involved in intestinal structure integrity.

Metabolic activity measurements revealed no inhibitory potential for ATX-II singularly and minor reductions for AOH, while the binary mixture reduced cell viability (resazurin metabolism, Figure 11, Supporting Information Figure S1). Of note, these effects were not mirrored at the protein level (Figure 11B) and did not reflect on the cell integrity (LDH release, Figure 11C).

To investigate the toxins’ immunomodulatory potential on colon epithelial cells HCEC-1CT, an inflammatory state was provoked by stimulation with IL-1β. qRT-PCR experiments revealed the potential of AOH to suppress IL-1β-induced mRNA increase of IL-8 and TNF-α, while ATX-II suppressed only TNF-α transcription. The reduced efficacy of the binary mixture in this regard suggests distinct mechanisms of action for the two *Alternaria* toxins (Figure 2). Of note, both AOH and ATX-II were previously reported to exert immunosuppressive effects in THP-1-derived macrophages and differentiated Caco-2 colon tumor cells.^27,44,45^ In addition, AOH was described to further enhance IL-1β-induced TNF-α mRNA transcription in Caco-2, albeit in higher concentration ranges. The reported effects were linked to inhibition of the NF-κB pathway.^44^ Here, using nontransformed intestinal cells, with the measurement of IL-8 secretion levels, we could demonstrate that both toxins can affect the proinflammatory response of IL-1β (5 h of incubation, Figure 3). Even though the literature regarding ATX-II is limited, a recent study in hormone-sensitive prostate cells reported AOH to suppress IL-1β gene transcription.^46^ Furthermore, Kowalska and colleagues found AOH to induce IL-6 at transcript and protein levels, an effect that could be partially linked to the estrogen-like capacities of AOH toward the estrogen receptor β (ER-β).^46^ The two cytokines IL-8 and TNF-α typically sustain the early phases of acute inflammation.^47^ Hence, we postulate that modulations by *Alternaria* toxins obtained even in short incubation times (Figures 2 and 3) might impact on the cytokines’ downstream signaling and further influence subsequent processes within the immune response.^47^ The timeline/kinetics of the toxins’ colonic absorption/metabolism is, in particular, relevant for any interpretation of the toxicological potential of food contaminants. AOH was previously reported to undergo rapid absorption and phase-II-metabolism yielding AOH-GlcA and AOH-S *in vitro*.^48^ However, if subjected to enterohepatic circulation, gut microbial β-glucuronidases could lead to exposure of the colonic epithelium to the parent compound again.^49^ This mechanism is known to participate in drug toxicity^50^ and was reported for structurally similar xenobiotic small molecules, such as triclosan,^49^ or endogenous estrogens.^51^ Hence, even assuming a low-concentration dietary intake (see Cell Culture and Experimental Layout section), this does not exclude the possibility of recurrent intestinal exposure to *Alternaria* toxins. ATX-II, however, is rapidly metabolized to ATX-I *in vitro* and *in vivo*. Thus, ATX-I was previously recovered in rat feces and urine.^21,52^ For the inflammatory cascade, inhibition of NF-κB activation in THP-1 cells was recently associated with the capacity to induce oxidative stress for ATX-II.^27^ In this light, it might be crucial to understand to what extent the biological activity of ATX-II can be attributed to long-term or short-term effects. Apart from the epoxide moiety, the two perylene quinones ATX-II and ATX-I share a planar scaffold, which accounts for the potential to synergistically activate AhR and this effect is independent of the stability of ATX-II.^31^ Moreover, AOH was also reported for its AhR-activating potency,^53^ and hence, the three toxins were suggested to act as major contributors to the overall AhR-activating capacity of complex *Alternaria* toxin mixtures.^31^

Numerous studies suggested AhR activation to interfere with the NF-κB pathway, postulating implications for inflammatory diseases and intestinal homeostasis.^8^ Immunostaining experiments revealed both *Alternaria* toxins, as single compounds and in combination, to activate the AhR pathway by enforcing AhR nuclear translocation in a noninflammatory setting, as well as under inflammatory stress (Figures 4 and 6). In support of the interpretation that AOH and ATX-II could activate the complete AhR pathway despite minor reductions in cell metabolic activity (Figure 11A), activation of CYP 1A1/1A2/1B1 isoforms by these toxins was recently suggested to be decoupled from the broad-ranging variety of resazurin-reducing enzymes within the cytosol and mitochondria, as shown by the CTB assay.^31,54^ Of note, as recently published, the AhR-activating potential of AOH and ATX-II seems to be tissue- and concentration-dependent and varies due to diverging kinetics according to the cell model.^31,55^ However, considering that the reference compound B[*a*]P showed similar behavior as the toxins in regard to AhR pathway activation and immunosuppressive capacities (Figures 2–4 and 6), we hypothesized a potential cross talk between the two signaling pathways. Indeed, NF-κB/p65 localization experiments revealed a suppressive effect of AOH on IL-1β-triggered nuclear translocation (Figure 7D). Similarly, urolithins, bacterial metabolites of ellagitannins similar in structure to AOH,^56^ were described to have immunosuppressive effects via the NF-κB pathway: at the molecular level, this was related to the interaction with their Nrf2-ARE pathway.^56^ Along this line, AhR pathway activation was suggested to interfere with the cellular oxidative stress response,^8^ when in turn, several feedback loops between the Nrf2-ARE pathway and NF-κB activation have been reported.^11^ Building on this, immunofluorescence imaging experiments were conducted to visualize the transcription factor Nrf2 (Figure 5), in addition to AhR and NF-κB. Accordingly, B[*a*]P (1 μM, AhR positive control), AOH (10 μM), ATX-II (1 μM), and binary combination incubations (1 h) enhanced nuclear/cytosolic ratios of Nrf2 (Figure 5). These results are in line with previous studies describing the capacity of AOH (10 μM) and ATX-II (5 μM) to induce oxidative stress via the Nrf2-ARE pathway in HT29 cancer cells^30,57^ and HCEC-1CT cells.^29^ Furthermore, AOH (10 μM) was recently reported to induce generation of ROS and modulate SOD1 gene expression and the protein level in human prostate cells. Besides, the toxin was found to suppress relative gene expression of the NF-κB subunit p65 (RelA), partly attributed to the endocrine capacity of AOH, in this particular *in vitro* model.^46^ Supportive of the cross talk between AhR and Nrf2, CH-22, known as AhR antagonist,^33^ showed the capability to suppress the Nrf2 nuclear/cytosol ratio for all substances tested (Figure 5). On this note, it was recently reported for urolithin A (Uro A) that expression of both Nrf2 and AhR is crucial for the chemicals’ potential to increase mRNA levels of Nrf2 and heme oxygenase 1 (HO-1) in colon explants from mice.^58^ Mechanistically, the toxins’ activating capacity toward Nrf2 could participate in their anti-inflammatory potential via the Nrf2/heme oxygenase 1 (HO-1) axis, as HO-1 itself and its enzyme products (such as CO) are known to suppress proinflammatory cytokines triggered by the NF-kB pathway and induce anti-inflammatory cytokines.^59^

Inflammatory intestinal disorders are accompanied by pro- and anti-inflammatory cytokines produced by intestinal cells and/or acting on them.^60^ Excessive inflammation is known to impact IECs by altering the epithelial barrier integrity, which is characterized by modified expression levels of tight junction proteins.^4^ For instance, Pujada and colleagues previously reported elevated expression of MMP-9 to modulate tight junction proteins and hence impact on the epithelial permeability in colitis-associated cancer.^61^ In HCEC-1CT cells, AOH and ATX-II suppressed IL-1β-induced MMP-2 mRNA transcription (dose-dependently for AOH) (Figure 2C) as single compounds, with reduced efficacy in combination. In turn, while MMP-9 transcription was induced by IL-1β, single-toxin exposure slightly enhanced this, yet not in the binary mixture (Figure 2D). Recently, AOH was reported to trigger both MMP-2 and MMP-9 mRNA transcription in mammary cells: however, this effect was biphasic and concentration-dependent; in fact, 10 μM AOH even slightly decreased MMP-9 mRNA relative transcription compared to solvent controls.^62^ In the same study, zymography experiments revealed decreased enzyme activity for both MMPs, which was linked to reduced wound-healing capacity and changes in adhesion. Simultaneously, the relative expression of vimentin, a regulator of epithelial-to-mesenchymal transition was suppressed at a low concentration and enhanced at 10 μM. Mechanistically, these observations were connected to the toxins’ immunomodulatory and oxidative stress-inducing capacities.^62^ Additionally, in relation to its potential to modify the membrane architecture in immune cells, AOH was recently reported to reduce LPS-induced Toll-like receptor 4 (TLR4) increase/recruitment in THP-1 -derived macrophages.^63^ Also, ATX-II was previously described to modify membrane fluidity and cell structure in HCEC-1CT, particularly in relation to increased oxidative stress and modification of the Nrf2 translocation profile.^29^ Hence, cell response to inflammation and oxidative stress strongly relates to cell morphology.^64^ IECs are recognized for their function as an essential barrier against toxins and pathogens. They fulfill their purpose by strictly regulating proteins of the apical junctional complex^65^ to form a “gate and fence” toward molecules.^66^ In our experimental setup, when inflammation was suppressed with Dex (1 μM), immunolocalization experiments (Figure 8) revealed a significant increase in Cldn 4, ZO-1, and integrin β1 cytosolic localization. This could be achieved also with incubation with ATX-II (1 μM) and to some extent in the mixture treatments (for ZO-1 and integrin β1). The efficacy of AOH was limited to integrin β1 (Figure 8). In line, we could postulate the immunomodulatory potential of *Alternaria* toxins to be downstream in the regulation of intestinal structural elements. In fact, bidirectional effectivity on epithelial barrier function was previously reported for both cytokines investigated in this study, among others. For example, proinflammatory cytokines, such as IL-1β, IL-8, and IFN-γ, were linked to reduced tight junction protein gene expression.^67^ TNF-α is known to play dual roles in inflamed microenvironments of IECs, leading to barrier defects on the one hand, yet contributing to wound healing on the other hand.^68^ Mechanistically, we could also describe how the response on tight junction proteins elicited by AOH and ATX-II (Figure 8) could be modulated by co-incubation with CH-22, indicating an involvement of the AhR pathway in these effects. Furthermore, AhR is known to participate in the colonic immune response and preservation of the epithelial barrier.^16^ This is in line with a recent report on AhR activation to impact epithelial barrier integrity, via PKC and p38MAPK,^69^ inflammatory signaling cascades often triggered simultaneously to NF-κB.^5^ Of note, AOH was previously suggested to induce DNA damage via p38MAPK and ATF2 signaling.^70^ Additionally, also the Nrf2-ARE and AhR signaling were recently identified to modulate colonic epithelial barrier function for the microbial metabolite Uro A.^58^ A multiplicity of AhR-related pathways potentially connect inflammation and epithelial barrier integrity at the intestinal level. Thus, apparent slight discrepancies between the mechanism of action of the positive control for AhR activation, B[*a*]P, and the two *Alternaria* toxins on several endpoints throughout this study could be interpreted also in light of previous studies, suggesting AhR downstream effects to be ligand-dependent.^71^ Indeed, several AhR ligands were described to vary in their extent, nature, and follow-up implications of AhR modulation in a tissue- and cell-type-specific manner.^72^ To gain an insight into the potential of AOH and ATX-II to affect cell structure in relation to changes in the experimental model, high-cell-density immunostaining experiments were also performed. ATX-II and combinatory exposure to AOH and ATX-II (10:1) enhanced ZO-1 cytosolic localization with or without IL-1β stimulation (Figure 9A–C,E–G). Moreover, a clearly visible alteration in the distribution of the scaffold protein could be observed (Figure 6D,H). Furthermore, ATX-II enhanced Ocln expression levels (Figure 10E,F). This is in line with a previous report on the AhR-activating compound β-naphtoflavone to support the reassembly of tight junction proteins such as ZO-1 upon chemical disruption in Caco-2/TC7 cells, as well as enhancement of Ocln and tricellulin proteins, found at cell–cell contacts.^69^ Considering other parameters that can potentially influence barrier integrity and tissue organization, our experiments revealed that AOH, but foremost ATX-II and the binary mixture, can significantly change HCEC-1CT cells’ morphology.

## Conclusions

Taken together, to the best of our knowledge, we are the first to report immunosuppressive capacities of both toxins AOH and ATX-II in a noncancerous colonic cell model. At the molecular level, these effects could be traced back to the activation of the aryl hydrocarbon receptor signaling and the Nrf2-ARE pathway. Moreover, we could show the two *Alternaria* toxins’ potential to modulate the expression of membrane-bound proteins necessary for epithelial barrier integrity and reconstruction. In this light, we could describe the toxins to impact several levels of the colonic immune response and crucial players within the epithelial organization during inflammation. In conclusion, both toxins require further attention as food contaminants, especially considering that the gut is prone to recurrent, or even chronic inflammatory stimuli throughout a lifetime.

## Supporting Information Available

Five hours of cell viability testing results; incubation and workflow chart; overview of immunofluorescence images of tight junction transmembrane proteins for respective incubation conditions; cell metabolic activity (CellTiter-Blue assay) after 5 h of toxin exposure and 3 h of concomitant IL-1β stimulation (Figure S1); schematic depiction of the incubation workflow for the different experiments carried out (Figure S2); representative immunofluorescence images of the cell membrane and tight junction proteins after 24 h of incubation (Figure S3) (PDF)
