# Supplementary figures and images for: Alternaria alternata Mycotoxins Activate the Aryl Hydrocarbon Receptor and Nrf2-ARE Pathway to Alter the Structure and Immune Response of Colon Epithelial Cells
Source: Chem Res Toxicol. 2022 Apr 11;35(5):731–49. doi: 10.1021/acs.chemrestox.1c00364 (PMC9115800; doi:10.1021/acs.chemrestox.1c00364)

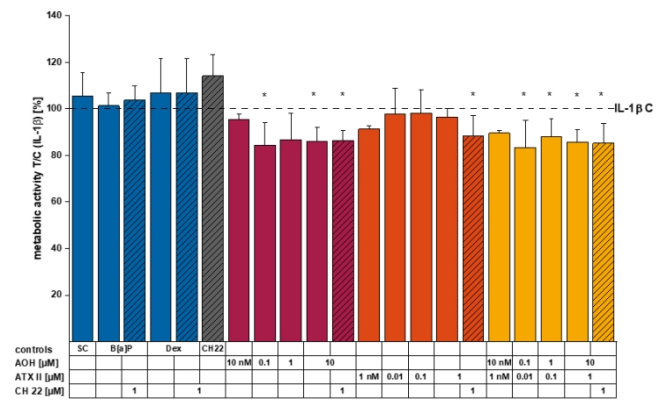

338x190mm (96 x 96 DPI)

Supplement: Supplementary file 1 — tx1c00364_si_001.pdf [file tx1c00364_si_001.pdf]

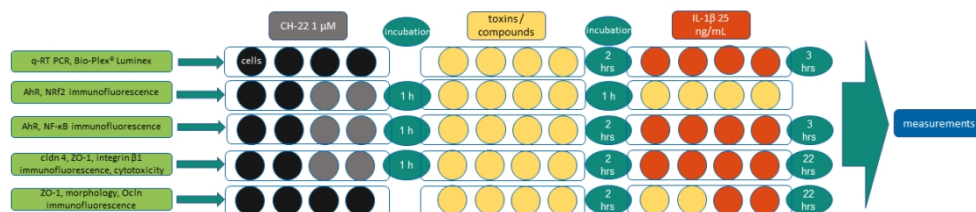

338x190mm (96 x 96 DPI)

Supplement: Supplementary file 2 — tx1c00364_si_002.pdf [file tx1c00364_si_002.pdf]

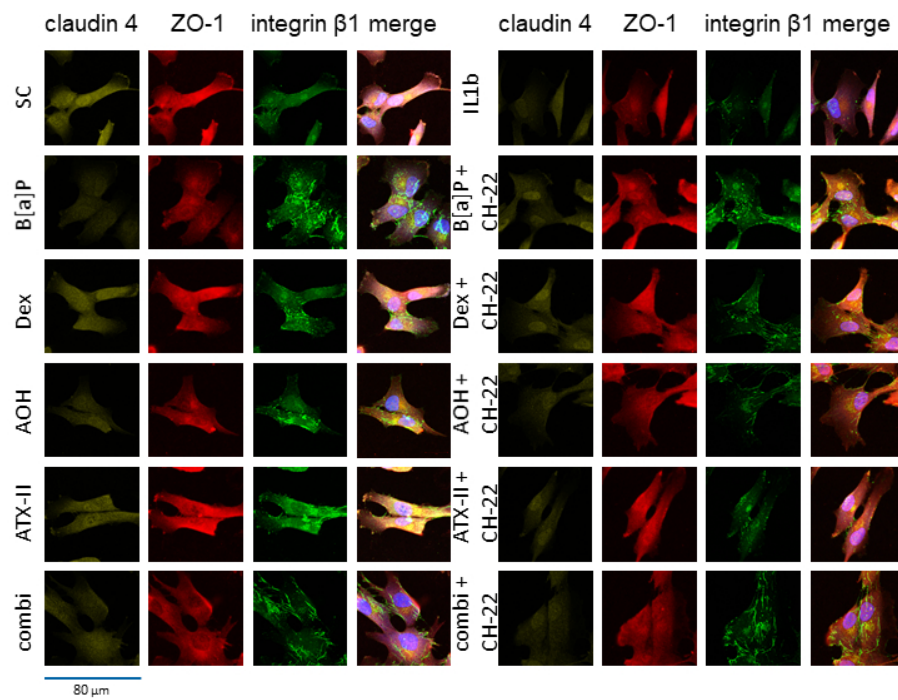

190x254mm (96 x 96 DPI)

Supplement: Supplementary file 3 — tx1c00364_si_003.pdf [file tx1c00364_si_003.pdf]
