# Supplementary material for: Alternaria alternata Mycotoxins Activate the Aryl Hydrocarbon Receptor and Nrf2-ARE Pathway to Alter the Structure and Immune Response of Colon Epithelial Cells
Source: Chem Res Toxicol. 2022 Apr 11;35(5):731–49. doi: 10.1021/acs.chemrestox.1c00364 (PMC9115800; doi:10.1021/acs.chemrestox.1c00364)
Supplement: Supplementary file 4 — tx1c00364_si_004.pdf [file tx1c00364_si_004.pdf]

Supplementary File 1

Alternaria alternata mycotoxins activate the aryl hydrocarbon receptor and Nrf2-ARE pathway to alter structure and immune response of colon epithelial cells

Julia Groestlinger[†], *Veronika* Spindler[‡], *Gudrun* Pahlke[†], *Michael* Rychlik[‡], *Giorgia* Del Favero[†,§,*], *Doris* Marko[†,*]

[†] Department of Food Chemistry and Toxicology, University of Vienna, Währinger Straße 38, 1090 Vienna, Austria

[‡] Chair of Food Analytical Chemistry, Technical University of Munich, Germany

[§] Core Facility Multimodal Imaging, Faculty of Chemistry, University of Vienna, Währinger Straße 38, 1090 Vienna, Austria

[*]Correspondance: doris.marko@univie.ac.at (D.M.) Giorgia.del.favero@univie.ac.at(G.D.F.)

Chemical Research in Toxicology

Table of Contents:

- 5 hours cell viability testing results
- Incubation & workflow chart
- Overview of immunofluorescence images of tight junction transmembrane proteins for respective incubation conditions

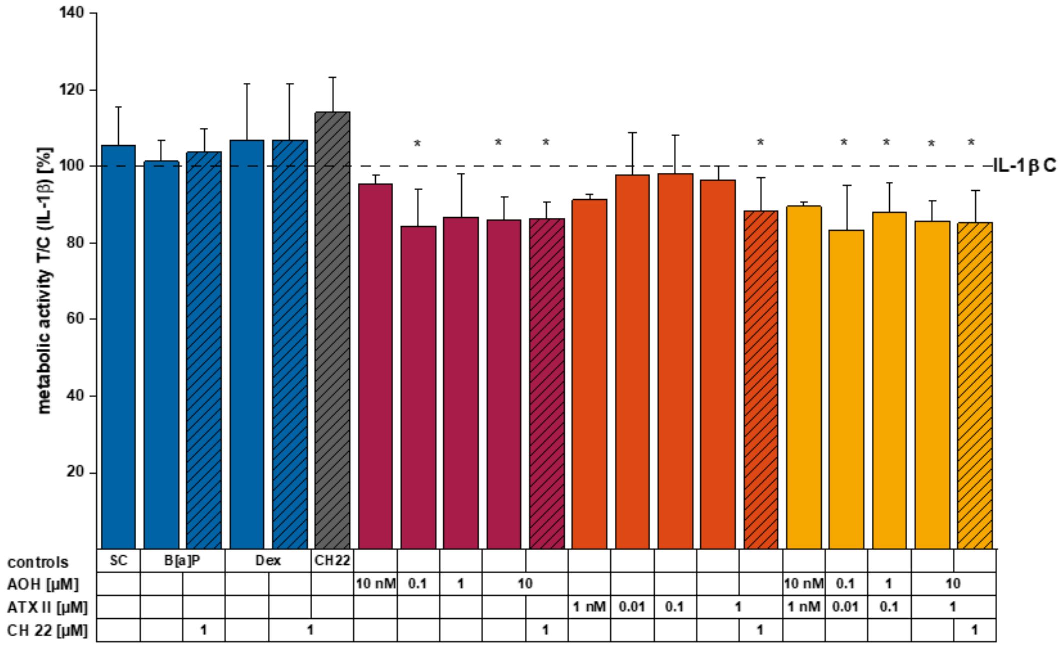

Supplementary Figure 1. Cell metabolic activity (CellTiter® Blue assay) after five hours of toxin exposure and 3 hours concomitant IL-1β stimulation. Data are presented as mean + SD of at least 3 individual experiments conducted in technical triplicates. Metabolic activity was normalized to stimulus control and significant differences compared to IL-1β control are marked as * (*$p<0.05$*) and were calculated applying Students' *t*-test.

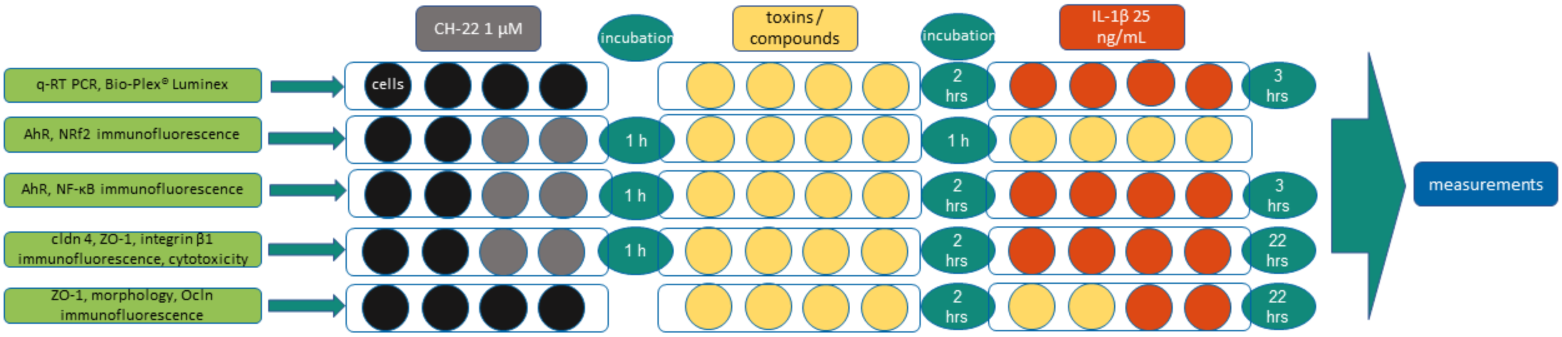

Supplementary Figure 2. Schematic depiction of the incubation workflow for the different experiments carried out. Cells were seeded for 48 hours prior to different incubation scenarios according to the respective experiment.

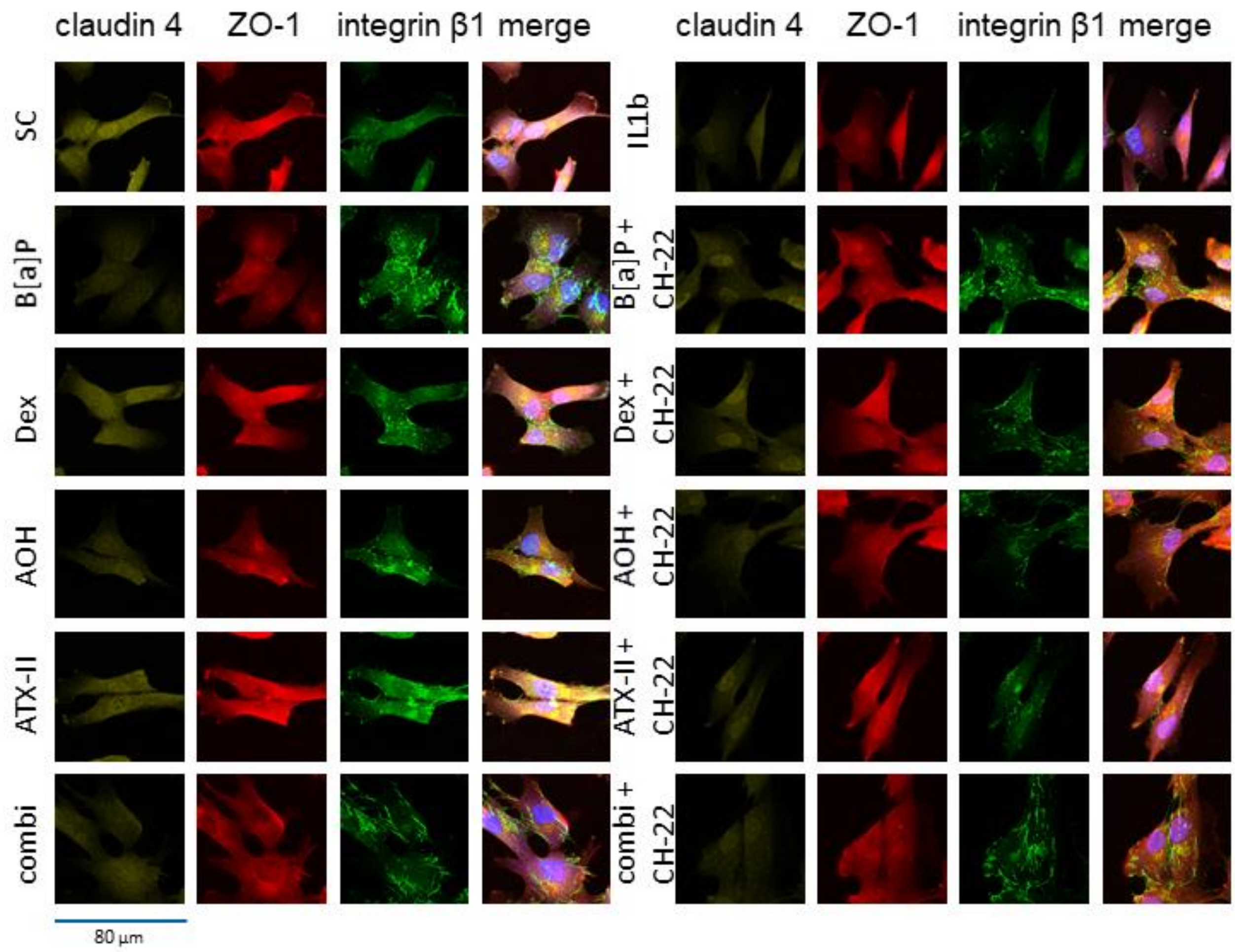

Supplementary Figure 3. Representative immunofluorescence images of cell membrane and tight junction proteins after 24 hours of incubation. Panel shows individual channels for each incubation condition.
